# Microfluidic–optical integrated portable platform with handheld pump for wash-free plasmonic detection of thrombin and SARS-CoV-2 spike protein

**DOI:** 10.1038/s41378-026-01381-3

**Published:** 2026-07-17

**Authors:** Da-In Kwon, Yeong-Eun Yoo, Jae-Ho Jin, Ji Hyo Park, Ga Eun Han, Kwanoh Kim, Jae Sung Yoon, Seong Min Kang, Do Hyun Kang

**Affiliations:** 1 Nano-Lithography & Manufacturing Research Center, Korea Institute of Machinery and Materials (KIMM), Daejeon, South Korea; 2 Department of Mechanical Engineering, Sogang University, Seoul, South Korea; 3 Neo Nanotech Co., Ltd., Daejeon, South Korea; 4 Department of Chemistry, Chung-Ang University, Seoul, South Korea; 5 Advanced Bioconvergence, University of Science & Technology, Daejeon, South Korea

**Keywords:** Engineering, Biosensors, Microfluidics

## Abstract

Point-of-care testing (POCT) requires rapid, quantitative results without the complexity of laboratory instrumentation. Conventional assays like ELISA and PCR are often limited by long processing times, multiple washing procedures, and a dependence on bulky, external power-consuming pumps. To address these challenges, we developed a fully integrated, palm-sized platform validated with two high-impact biomarkers: thrombin, a critical regulator of blood coagulation for cardiovascular and renal diagnostics, and the SARS-CoV-2 spike protein, a primary antigen for rapid viral screening. The platform’s innovation lies in its user-independent, electricity-free fluidic operation, featuring a manual suction pump with a compression spring that ensures high reproducibility (CV < 4.5%). Additionally, a serpentine microchannel design was incorporated to promote Dean flow-induced inertial mixing, accelerating aggregation kinetics for a true wash-free plasmonic assay. Our system provides real-time quantitative detection within 5 min, achieving sub-nanomolar limits of detection (LoD: 0.5 nM for thrombin and 0.4 nM for spike protein). Wireless connectivity allows for immediate data visualization on smartphones. By bridging the gap between sophisticated laboratory analysis and portable, manual operation, this platform offers a robust and scalable solution for decentralized clinical diagnostics.

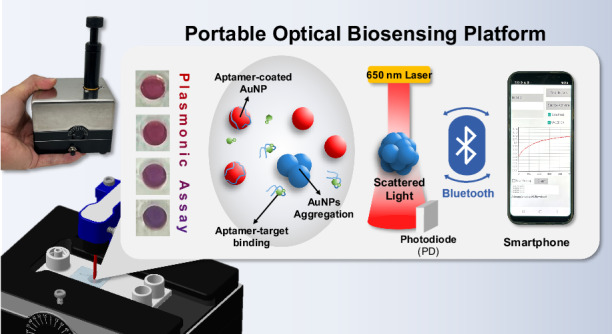

## Introduction

Portable integrated biosensor platforms are a promising tool for realizing upcoming point-of-care testing systems that provide rapid diagnostic results without being constrained by location or the need for skilled personnel. Such an integrated platform can be utilized in diverse fields, including bedside healthcare^1–4^, infectious disease management^5–7^, environmental monitoring^8^, food safety investigation^9^, and drug screening^10^. In particular, developing countries, where medical infrastructure is often insufficient, require portable integrated platforms that do not depend on expensive diagnostic instruments^5,7,9,11^. The COVID-19 pandemic has also further emphasized the need for rapid, sensitive, and field-deployable diagnostic platforms^12^. Recent developments in microfluidic devices, biorecognition elements, and miniaturized optical or electrical instruments have significantly improved the sensitivity, specificity, cost, convenience, and portability of biosensing systems^13–16^. For real-world deployment, such portable platforms should support simple and rapid assays, require small sample volumes, and minimize risk of cross-contamination^17,18^. A straightforward microfluidic control strategy for liquid bio-samples is required to replace bulky and high-power external pumps. Moreover, connectivity with smartphones or PCs for real-time data processing and visualization is crucial for field usability^1,2,5,18^. To meet these demands, a novel, fully integrated biosensor platform is needed, combining microfluidics, specific biorecognition, optical detection, and digital communication into a single compact system.

The nanoparticle aggregation-based assay using gold nanoparticles (AuNPs) and aptamers can be an excellent sensing strategy for building the integrated biosensor platform. AuNPs have been used in various bioassays due to their specific optical property called surface plasmon, as well as their long-term stability and biocompatibility^19–26^. Aptamers, which are synthetic oligonucleotides, have gained significant attention due to their selective affinity for specific target molecules^27–30^. Aptamers are considered more stable and cost-effective than conventional probes such as antibodies. In this study, AuNPs were synthesized using the classical Turkevich method^31–33^, which was selected for its robust reproducibility and its ability to yield citrate-stabilized surfaces ideal for spontaneous aptamer adsorption. Crucially, the citrate-stabilized AuNPs and aptamers have a unique interaction that can be utilized for a rapid and wash-free bioassay, allowing convenient detection of diverse target samples such as proteins^32,34,35^, bacteria^36,37^, viruses^38^, and small molecules^39–41^. Pristine aptamers inherently prevent AuNPs from aggregating, even at high salt concentrations, by adsorbing and stabilizing the surfaces of the AuNPs. In contrast, target-bound aptamers lose their adsorption properties to the AuNPs surface, resulting in significant aggregation of AuNPs under high salt concentrations. Such AuNPs aggregation mediated by aptamers and their targets instantly causes a substantial plasmonic response, which can be easily monitored by optical scattering or absorbance measurements.

**Scheme 1:**
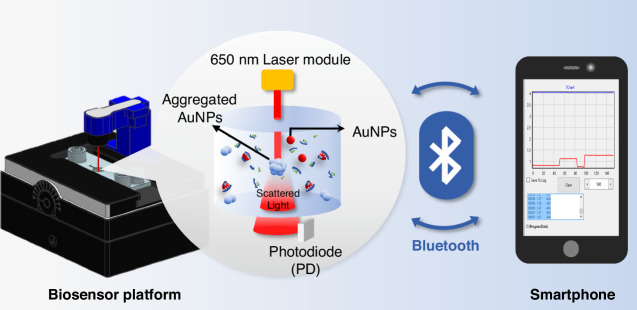
**Portable integrated microfluidic platform for AuNPs aggregation-based bioassay**. Schematic illustration of the platform using AuNPs and aptamers for aggregation-based detection with smartphone readout

In this study, we devised a portable integrated biosensor platform for convenient point-of-care testing applications. As illustrated in Scheme 1, our platform utilizes microfluidic systems for handling small volumes of reagents with high reproducibility. A mountable microfluidic chip, consisting of serpentine microchannels, is designed for aggregation-based bioassays using AuNPs and aptamers. We also constructed a user-independent manual handheld pump that can generate flow inside the microchannel by creating negative pressure between inlets and outlets with a single push. Additionally, the developed platform includes a miniaturized optical detection module consisting of a 650 nm laser emitting diode and a photodiode for detecting the light scattering signal of the aggregation-based bioassay. Such miniaturization reduces the overall size of the platform to palm-size. Furthermore, we integrated an electric circuit into the platform for interpreting, recording, and displaying the light scattering intensity corresponding to target biomolecule concentration. The signal can also be transmitted to nearby smartphones and PCs through practical wireless Bluetooth communication.

We applied our developed platform to detect two critical biomarkers: thrombin and the SARS-CoV-2 spike protein. Thrombin is a key protein in the blood coagulation process, responsible for activating platelets and converting fibrinogen to fibrin, thereby promoting clot formation^42^. Elevated levels of thrombin in plasma or urine indicate cardiovascular diseases or urinary tract disorders, respectively^43–45^. Thus, monitoring thrombin levels in these fluids can help assess the efficacy of treatments and track disease progression. In parallel, SARS-CoV-2 is the virus that triggered the recent global pandemic, leading to widespread illness, significant mortality, and profound social and economic impacts worldwide. Developing diagnostic kits that target the abundant spike proteins on the surface of the SARS-CoV-2 virus is an effective strategy for achieving high sensitive detection of the dangerous virus^46^. Utilizing high-affinity aptamers against thrombin and the SARS-CoV-2 spike protein, we implemented an aptamer-mediated AuNPs aggregation assay within our integrated platform. Because the aggregation reaction occurs directly upon target binding, the assay does not require washing or reagent exchange steps. While lateral flow assays (LFAs) are widely used for rapid testing due to their simplicity, they often provide only qualitative or semi-quantitative results with limited sensitivity. Conversely, conventional assays such as ELISA or PCR offer high precision but typically involve multiple complex processing steps and specialized equipment. The proposed system bridges this technological gap by enabling direct, real-time monitoring of AuNP aggregation in a user-independent, quantitative format, achieving high fidelity without the need for additional handling or external power.

Importantly, our platform introduces three distinctive features that differentiate it from previously reported handheld plasmonic systems. First, the assay operates in a true one-step, wash-free format, enabling sample-to-result analysis in less than 5 min without sequential reagent handling. Second, fluid transport is driven by a purely manual suction pump, eliminating the need for electrical pumps or external power sources for liquid handling while maintaining high chip-to-chip reproducibility (CV < 5%). Third, the serpentine microchannel design promotes Dean flow-induced inertial mixing, which accelerates the aggregation reaction and enables rapid signal generation even under manual actuation conditions. This simplified workflow reduces operational complexity. Compared with benchtop spectrometers and pump-based systems, the compact configuration decreases instrument footprint while integrating optical detection and fluid handling. Furthermore, the modular microfluidic architecture allows potential adaptation to multiplexed formats by functionalizing separate channels with distinct aptamer-AuNPs conjugates.

## Materials and methods

### Materials

The chemical reagents, such as gold (III) chloride trihydrate (HAuCl₄·3H₂O), trisodium citrate, sodium chloride, and phosphate-buffered saline (1× PBS, pH 7.4), were purchased from Sigma-Aldrich Chemicals. Thrombin from human plasma (lyophilized powder), synthetic urine, and albumin from human serum were also obtained from Sigma-Aldrich. SARS-CoV-2 (COVID-19) spike recombinant protein was purchased from ProSci. Previously reported aptamers with affinity for thrombin and SARS-CoV-2 spike protein were ordered from Bioneer, Daejeon, South Korea. The sequence of the thrombin-binding aptamer (TBA) is AGT CCG TGG TAG GGC AGG TTG GGG TGA CT (5’ to 3’, 29 mer)^32,47^, and the sequence of the spike protein aptamer is CAG CAC CGA CCT TGT GCT TTG GGA GTG CTG GTC CAA GGG CGT TAA TGG ACA (5’ to 3’, 51 mer)^46,48^. Both aptamers have an amine moiety with a six-carbon linker at their 5’ end site. Aliquoted stock solutions of the aptamers and thrombin were prepared by dissolving in 1× PBS and stored at −20 °C to ensure the preservation of bioactivity. The urine and albumin, employed as control substances, were stored at 4 °C.

### Synthesis and characterization of gold nanoparticles (AuNPs)

AuNPs were synthesized by using the Turkevich method^31–33^ (Scheme S1). Initially, HAuCl₄·3H₂O (153 µL, 10% w/w) was mixed with 100 mL of deionized (DI) water in an Erlenmeyer flask. The flask was then placed in a bath filled with Lab Armor beads (Lab Armor) to ensure uniform heat distribution. Stirring was performed at 800 rpm using a magnetic stirrer, and the temperature inside the flask was adjusted to 98 ± 1 °C. Subsequently, sodium citrate solution (2 mL, 194 mM) was added to the mixture. Sodium citrate acts as a reducing agent, converting gold ions in the solution to gold atoms, which then nucleate and grow into nanoparticles. This transformation is indicated by the solution’s color change from yellow to gray and finally to dark red, signifying the formation of AuNPs. The reaction was allowed to proceed for approximately 20 min after the addition of sodium citrate. Following synthesis, the AuNPs colloid solution was wrapped in aluminum foil to protect it from light and was stored at 4 °C. The optical properties of the synthesized AuNPs were characterized using a UV-vis spectrometer (Shimadzu UV-2600, Fig. S1A). Z-average diameter and zeta potential of the NPs were measured by Nano Particle Size Analyzer (Malvern Nano ZSP) (Fig. S1B, C). Particle morphology was further confirmed by scanning electron microscopy (SEM, TOPCON SM-350). The storage stability of AuNPs was monitored for 180 days using a UV-vis spectrometer (Fig. S2).

### Nanoparticle aggregation-based thrombin assay using AuNPs and aptamers in microtube

#### Optimization of TBA concentration

TBA concentration for the AuNPs aggregation-based thrombin assay was optimized by the following procedure. To a 170 µL solution of AuNPs in a 1.5 mL microtube, 30 µL of TBA at various concentrations (0–10 µM in 1× PBS) was added and pipetted. After 1 min of incubation, 230 µL of NaCl (0.1 M) was added to the AuNPs-TBA solution and pipetted. After 5 min of incubation, 100 µL of the AuNPs suspension was loaded into a coverwell chamber (Thermo Fisher Scientific), and the scattering intensity was measured under dark conditions using a fiber-optic UV-vis spectrometer (Ocean Optics FLAME). A 638 nm LED light source was used for excitation, and the scattered light was collected by a photodetector positioned at a 45° angle relative to the incident light (Fig. S3). The optimized TBA concentration was 4.59 µM, which was the minimum required to suppress nonspecific NaCl-induced aggregation of AuNPs (Fig. S4).

#### Optimization of NaCl concentration

NaCl concentration was similarly optimized by the following procedure. To a 170 µL solution of AuNPs in a 1.5 mL microtube, 30 µL of TBA (4.59 µM in 1× PBS) was added and pipetted. After 1 min, 30 µL of thrombin (276 nM) was added to the AuNPs-TBA solution and pipetted. For the control experiment, an equivalent volume of pristine 1x PBS buffer (pH 7.4) was used instead of the thrombin solution. After 5 min, 230 µL of NaCl at various concentrations (0.1 M, 0.5 M, and 1.0 M) were added to the AuNPs-TBA solutions with and without thrombin. The samples were then incubated for an additional 5 min before measuring the scattering light intensities using a fiber-optic UV-vis spectrometer. The optimized NaCl concentration was set to 0.1 M, which was sufficient to induce target-specific aggregation while minimizing nonspecific salt-driven aggregation (Fig. S5). At higher concentrations, both target and control solutions aggregated simultaneously leading to reduced relative scattering ratios and diminished assay specificity.

#### Optimization of pH

The assay pH was optimized using the following procedure. Reagent solutions were prepared in 10 mM MES buffer (pH 5.7), 1× PBS (pH 7.4), and 10 mM Tris buffer (pH 8.7). To 170 µL of AuNPs solution in a 1.5 mL microtube, 30 µL of TBA (4.59 µM) was added and mixed. After 1 min, 30 µL of thrombin (276 nM) was introduced into the AuNPs-TBA solution. For the control experiment, an equivalent volume of pristine PBS (pH 7.4) was added instead of thrombin. After 5 min, 230 µL of 0.1 M NaCl was added, and the samples were further incubated for 5 min before scattering intensities were recorded using a UV-vis spectrometer. Based on these measurements, the optimal assay pH was determined to be 7.4 (Fig. S6).

#### AuNPs aggregation-based assay in microtube

Using the optimized conditions (4.59 µM TBA, 0.1 M NaCl, pH 7.4), the scattering intensity of AuNPs with and without thrombin was monitored. To 170 µL of AuNPs solution in a 1.5 mL microtube, 30 µL of TBA (4.59 µM in 1× PBS) was added and mixed. After 1 min, 30 µL of 1× PBS containing thrombin (276 nM) or buffer only (control) was introduced and mixed. After 5 min, 230 µL of 0.1 M NaCl was added, and the samples were further incubated for 5 min. Scattering intensities were then recorded using a fiber-optic UV-vis spectrometer with the photodetector positioned at a 45° angle.

### Development of a portable integrated optical sensor platform

#### Platform housing and supports for internal parts

The components of the portable integrated optical sensor platform are depicted in Fig. 2a. The platform housing and supports for internal parts were designed using CAD software, specifically AutoCAD (Autodesk, USA) and NX UG (Siemens PLM Software, Germany). The facilities of the Korea Institute of Machinery and Materials (KIMM) and SD Design (Daejeon, South Korea) were utilized for the precise machining processes required for manufacturing the platform housing and supports, including milling, drilling, bending, and wire cutting. The top face of the housing has a space for sliding and mounting the microfluidic chip and manual micropump, positioning the microfluidic chip between the light source and photodetector.

#### Optical measuring parts

For the optical measuring parts, a 650 nm laser emitting diode (Dotdot Laser, 3 V, 6 mm, Jenomall, South Korea) and a photodiode (Micro 1206 SMT, 040–111–411, Advanced Photonix, USA) were chosen as the light source and photodetector, respectively (Fig. 2d). The laser light source was fixed at the top part of the housing, while the photodetector was located at the bottom part of the housing. The distance between the laser light source and the center of the microfluidic chip viewing chamber is 18.75 mm, while the distance between the laser photodiode and the center was 11 mm. A 1.5 mm diameter and 3.0 T thickness optical aperture was attached to the face of both the laser diode and photodiode to reduce noise from surrounding lights. The optical aperture was made by wire cutting the stainless-steel (SUS) film.

#### Printed circuit board (PCB) and programs

The PCB and embedded program for real-time sensor signal processing and wireless Bluetooth signal transmission were developed by ES2 (Daejeon, South Korea) (Fig. 2e). The electric elements of PCB, such as MCU (CC254X), Bluetooth Low Energy (BLE) module, power switch, DC-DC converter, and Li-Po battery, were purchased from Mouser Electronics (Fig. S7). The PCB and embedded program can turn on and off the laser diode, record the voltage (VPD) on the photodiode (photodetector), and transmit the VPD value to the connected PC program or smartphone applications (Video S1). Video S1 illustrates the overall process for operating the platform, including a concise real-time signal test. The PCB is located inside the bottom part of the housing, along with a rechargeable battery. The PC program and smartphone application for sensor signal receiving and graphical user interface were also built by ES2 (Daejeon, South Korea) using RAD Studio (Embarcadero, USA), a cross-platform framework.

### Developments of miniaturized microfluidic system

#### Manual suction pump

The suction pump for microfluidic chip operation was designed to generate flow without reverse flow after being connected to the outlet of the microfluidic chip, as shown in Fig. 3a. The pump consists of a plunger, push-type button, compression spring, ball-type check valve, and diaphragm-type check valve. The dimensions of the compression spring are 9 × 35 mm (K48693256, Navimro, Korea). The diaphragm-type check valve (80505, Qosina) has a female Luer lock to form a firm and leak-proof Luer-lock connection with the outlet of the microfluidic chip. The other parts of the pump were manufactured through an in-house, precise manufacturing process at KIMM and SD Design.

#### Microfluidic mixing chip

The microfluidic chip consists of two reservoirs (inlets), an outlet, serpentine microchannels, and a view chamber, as shown in Fig. 4a. One of the two inlet reservoirs is used for adding the AuNPs-TBA solution and the bio sample solution, while the other reservoir is used for the NaCl solution. Two serpentine microchannels are designed for sequential mixing processes: (i) citrate stabilization, maintaining colloidal dispersion of citrate-capped AuNPs; (ii) aptamer adsorption, stabilizing AuNPs through aptamer adsorption on the nanoparticle surface; (iii) target binding, weakening the stabilizing interaction between aptamers and AuNPs; and (iv) NaCl-induced aggregation, triggered by electrostatic screening and leading to AuNP aggregation in the presence of the target. The channel width and height are 400 μm and 400 μm, respectively. At the end of the serpentine channel, the viewing chamber was built for the optical measurement of light scattering intensity from aggregated AuNPs. It was intentionally designed for the optical path passing through the viewing chamber to penetrate to avoid optical noise from the rough surface of the 3D-printed structure. The depth and diameter of the viewing chamber are 2.45 mm and 7.2 mm, respectively. Approximately 100 µL of solution can fill the viewing chamber. The outlet has a screw thread on its outer wall for a firm and leak-proof connection with the female Luer lock interface of the manual suction pump. The microfluidic chip was designed with NX UG, CAD program and fabricated using the following process. The upper part of the microfluidic chip was printed using a Form 3 stereolithography (SLA) 3D printer (Formlabs, USA). Formlabs’s clear resin V2 was used, and the printing angle was set to 45 degrees. The upper part was then sealed using a transparent adhesive-PE film with a thickness of 0.15 T (3 M) to prevent fluid leakage (Fig. S8).

### Detection of thrombin and SARS-CoV-2 spike protein using portable integrated optical sensor platform

The microfluidic chip was mounted onto the portable platform, and a manual suction pump was connected to the outlet via a Luer lock, as shown in Fig. 2a. For the detection of thrombin and SARS-CoV-2 spike protein, 170 µL of AuNPs solution was first added to one inlet reservoir, followed sequentially by 30 µL of target-specific aptamer solution (4.59 µM) and 30 µL of the target protein (thrombin or spike protein, diluted in 1× PBS). Simultaneously, 230 µL of 0.1 M NaCl was loaded into the second inlet reservoir to match the total reagent volume. The suction pump was then attached to the outlet and manually activated to initiate fluid flow through the microfluidic channel (Video S2). Video S2 provides a visual overview of the experimental protocol, illustrating each step of the process. As the solutions progressed through the microfluidic channel, they mixed and flowed into the viewing chamber, where they were vertically irradiated with a 650 nm laser. The resulting scattering signal, induced by target-specific AuNPs aggregation, was detected in real time as a voltage output (VPD) by a photodiode and custom PCB circuit. The VPD signal was then transmitted via Bluetooth to a connected PC or smartphone application for quantitative analysis. Various concentrations of thrombin (0.25–16 nM) and spike protein (0.1–3.4 nM) were tested to determine their respective dynamic ranges and limits of detection. The selectivity was evaluated by conducting control experiments using avidin and human serum albumin at the maximum concentration of each target analyte (16 nM for thrombin and 1.7 nM for spike protein), replacing the target biomolecules. For the target quantification, VPD_final/VPD_initial was calculated, where VPD_final and VPD_initial represent the final and initial values of VPD during the detection experiment.

## Results and discussions

### Detection of AuNPs aggregation by light scattering measurement

We utilized light scattering measurement to achieve sensitive protein detection based on nanoparticle aggregation. The alteration in plasmonic resonance due to AuNPs aggregation can be monitored using both transmission (or absorbance) and scattering measurements. Between the two, measuring variations in scattered light at an oblique viewing angle relative to the incident light offers advantages compared to transmission measurements at an angle conducted along the light source axis.

The intensity of scattered light is highly sensitive to the degree of nanoparticle aggregation. Specifically, 13 nm diameter AuNPs, which are much smaller than the wavelength (650 nm) of the laser light source, exhibit minimal Rayleigh scattering. Upon target-induced aggregation, the effective size of particle aggregates increases significantly, resulting in a transition to strong Mie scattering. This transition from Rayleigh to Mie scattering produces a substantial increase in scattered light intensity, enabling highly sensitive optical detection of nanoparticle aggregation^49–51^. Notably, in the Rayleigh regime, the scattering intensity is proportional to the sixth power of the particle aggregates diameter. Such light scattering is less affected by sample opacity or background interference, providing a convenient “turn-on” sensor signal correlates with the target concentration^49–51^. Prior to the development of our portable platform, we exploited a preliminary scattering measurement setup utilizing fiber-optic components to optimize the assay conditions, as shown in Fig. S3.

### AuNPs aggregation-based thrombin assay using aptamer

**Fig. 1:**
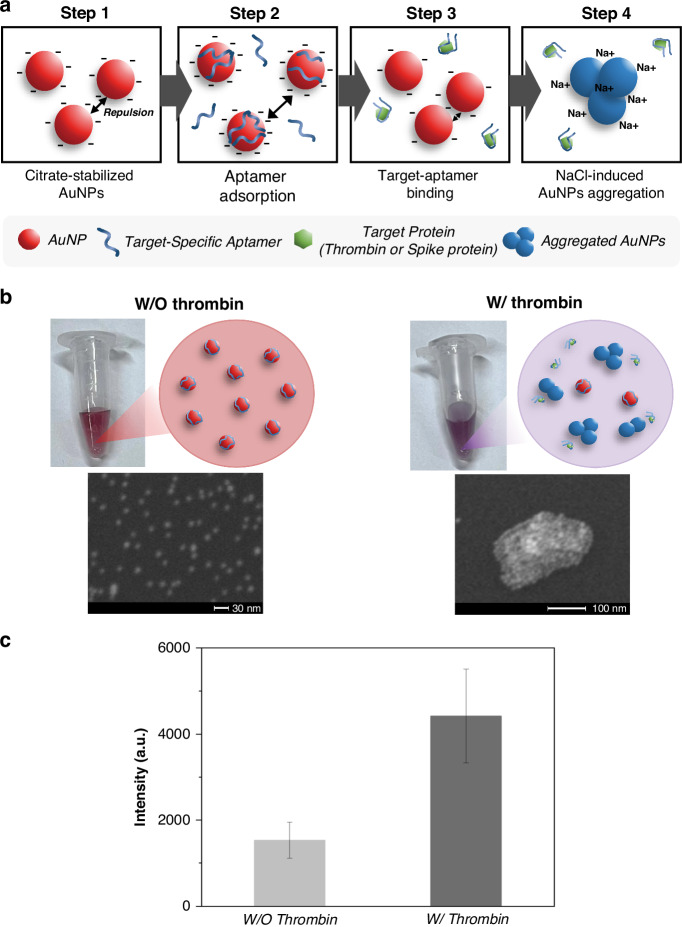
Thrombin detection using aptamer-mediated AuNPs aggregation. **a** Step-by-step schematic of the AuNPs aggregation assay mechanism: (Step 1) Citrate-stabilized AuNPs maintaining dispersion via electrostatic repulsion (indicated by negative charge symbols, -); (Step 2) Enhanced stabilization through aptamer adsorption; (Step 3) Target–aptamer binding and subsequent dissociation of aptamers from the AuNP surface; (Step 4) Salt-induced aggregation of unprotected AuNPs through electrostatic screening by Na^+^ ions, leading to a visible color change. **b** Photographs and SEM images of AuNPs before and after thrombin addition. **c** Scattering intensity showing increased signals in the presence of thrombin (16 nM) using a fiber-optic UV-vis spectrometer

We conducted AuNPs aggregation-based thrombin assay in a stepwise manner, as illustrated in Fig. 1a. Citrate-stabilized AuNPs were first synthesized and characterized to confirm their colloidal stability. The resulting AuNPs exhibited a distinct plasmonic peak at 519 nm with an optical density of approximately 1.3 (Fig. S1A). The plasmonic peak positions exhibited minimal variation among three separate synthesis batches, demonstrating robust batch-to-batch consistency. The diameters of AuNPs were approximately 13 nm and 24 nm, as determined by SEM image analysis and dynamic light scattering (DLS) measurements, respectively (Figs. 1b, S1B). The surface charge of AuNPs was determined to be −35 ± 3 mV by zeta potential analysis, indicating good colloidal stability due to the coating with negatively charged citric acid (Fig. S1C). Additionally, the plasmonic peak of AuNPs remained unchanged at 518 nm for the first 7 days and exhibited only a slight redshift to 520 nm after 180 days, demonstrating excellent long-term colloidal and plasmonic stability under refrigerated conditions (Fig. S2).

From a colloidal stability perspective, the stable dispersion of citrate-capped AuNPs can be rationalized by DLVO theory^52^, which describes the balance between van der Waals attraction and electrostatic double-layer repulsion in colloidal systems. The measured zeta potential (−35 ± 3 mV) indicates strong electrostatic stabilization of the nanoparticles. Upon aptamer adsorption, additional steric stabilization is introduced, further maintaining nanoparticle dispersion. However, target binding reduces effective surface passivation, and subsequent NaCl addition screens electrostatic repulsion, lowering the interparticle energy barrier and promoting aggregation. As a result, interparticle plasmon coupling occurs between neighboring nanoparticles, leading to a redshift and broadening of the localized surface plasmon resonance (LSPR) band. This aggregation-induced plasmonic coupling produces a visible color transition from red to purple and simultaneously enhances optical scattering signals. This target-mediated destabilization forms the physicochemical basis of the wash-free detection mechanism.

We further investigated the optimal concentrations of TBA and NaCl, and pH for the nanoparticle aggregation-based thrombin assay. The optimal TBA concentration of 4.59 µM was required to maintain colloidal dispersion after the replacement of citric acid coating on AuNPs surface and the addition of 0.1 M NaCl (Fig. S4). TBA concentrations below 4.59 µM resulted in incomplete surface passivation, leading to nanoparticle aggregation and a color shift to purple, while higher concentrations yielded no further stabilization benefit and were considered inefficient in terms of reagent usage. These results indicate that appropriate aptamer surface coverage is essential to maintain nanoparticle stability while preserving the ability to undergo target-triggered aggregation.

With respect to NaCl concentration, 0.1 M NaCl provided the highest signal difference between the positive (with thrombin) and negative (without thrombin) controls, although the concentrations higher than 0.1 M increased non-target-specific signals in the negative control (Fig. S5). This behavior can be attributed to excessive electrostatic screening at high ionic strength, which lowers the energy barrier for nanoparticle aggregation even in the absence of the target.

The effect of pH on thrombin-aptamer interaction and subsequent nanoparticle aggregation was also evaluated. Among thrombin solutions having pH 5.7, 7.4, and 8.7, the strongest aggregation response and color change occurred at neutral pH 7.4, likely due to optimal structural stability of thrombin and efficient binding with the aptamer (Fig. S6). In contrast, under acidic (pH 5.7) or alkaline (pH 8.7) conditions, reduced aggregation was observed, possibly due to conformational instability of both thrombin and the aptamer or changes in electrostatic repulsion between the assay components.

Using optimized reagent conditions and a scattering intensity measurement setup, we evaluated the optical response of TBA-functionalized AuNPs upon exposure to thrombin. As expected, thrombin induces significant aggregation of TBA-coated AuNPs, whereas AuNPs without thrombin maintain their stability against aggregation. SEM image shows that the TBA-coated AuNPs exposed to thrombin were aggregated, while the AuNPs without thrombin remained isolated, exhibiting a noticeable diameter of approximately 13 nm (Fig. 1b). The size of the aggregated AuNPs exceeded 150 nm, more than a tenfold increase. The increase in nanoparticle size due to aggregation also alters its plasmonic resonance property. The AuNPs with thrombin changed their color from red to purple, indicating a shift in plasmonic resonance properties. Figure 1c shows the scattering intensity of TBA-functionalized AuNPs upon thrombin exposure. In the absence of thrombin, the nanoparticles remained stable, resulting in relatively low scattering intensity. When thrombin was introduced, aptamer binding triggered nanoparticle aggregation, leading to a substantial increase in scattering intensity, consistent with the observed optical and morphological changes.

### Development of portable integrated optical sensor platform

**Fig. 2:**
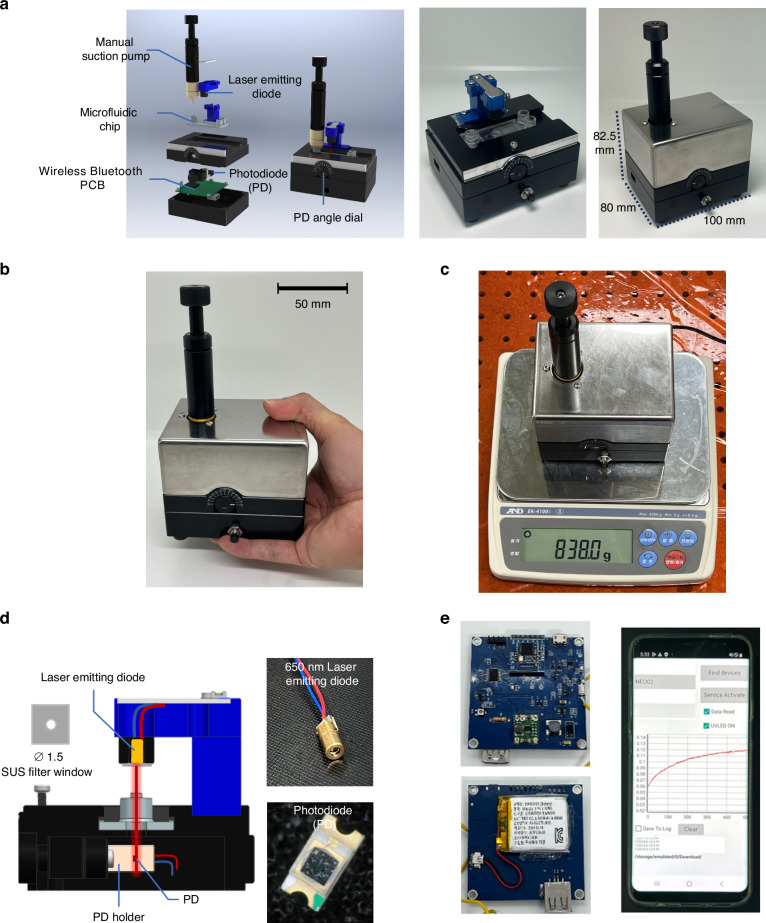
Design and components of the portable integrated microfluidic platform. **a** Schematic and photographs of the platform with the manual suction pump, microfluidic chip, laser diode, photodiode, and PCB. **b**, **c** Photograph of the palm-sized, portable device (80 × 100 × 82.5 mm; 838 g). Scale bar: 50 mm. **d** Cross-sectional view and optical components for scattering detection. **e** Bluetooth PCB and smartphone app displaying real-time signals

In this study, we developed a portable integrated biosensor platform for rapid, wash-free nanoparticle aggregation bioassays using gold nanoparticles (AuNPs) and aptamers. The platform integrates an optical module for real-time scattering measurements, a printed circuit board (PCB) for onboard signal processing, and a microfluidic unit with a disposable chip and manual suction pump (Fig. 2a). Excluding the suction pump, the device measures depth 80 × width 100 × height 82.5 mm (height 165 mm with pump) and weighs 838 g, making it palm-sized and easily portable (Fig. 2b, c). The overall device size falls within the size range of portable point-of-care diagnostic systems. For comparison, the commercially available portable analyzer, the Abbott i-STAT system (a portable device and separate reader measuring approximately 76 × 64 × 240 mm)^53^ and the Abbott ID NOW molecular diagnostics platform (approximately 250 × 170 × 130 mm)^54^, represent typical configurations for distributed diagnostic devices. While slightly larger than a small portable analyzer, this platform uniquely integrates passive microfluidic liquid drive, optical detection, and wireless communication into a single device, creating a standalone biosensing system suitable for point-of-care diagnostics without the need for bulky laboratory equipment.

The optical module consists of a 650 nm laser diode as the excitation source and a photodiode with a 100–300 nm visible detection range (Fig. 2d). To reduce background noise, each element was fitted with a 1.5 mm aperture, and a screen cover was added to block ambient light. This configuration enhanced sensitivity by minimizing interference and enabling precise detection of nanoparticle scattering signals. The photodiode alignment was adjustable from −61.5° to +61.5° relative to the laser beam, allowing flexible configurations for transmission or scattering detection. Detection at 61.5° provided the highest signal-to-noise ratio (Fig. S9) and was adopted for subsequent measurements. The PCB, located at the base of the platform, integrates an MCU, BLE module, power switch, DC-DC converter, and a Li-Po battery, enabling real-time monitoring and wireless communication with PCs or smartphones (Figs. 2e, S7). The accompanying software and mobile application display the photodiode voltage (V_PD_) in real time as both numeric readouts and time-series graphs (Fig. 2e and Video S1). Raw V_PD_ values are presented without smoothing or averaging, as unprocessed data more accurately capture the assay response. The PCB-powered system supports up to 48 h of continuous operation on a single battery charge and requires no coding or calibration by the user.

### Development of hand-operated suction pump and microfluidic system

**Fig. 3:**
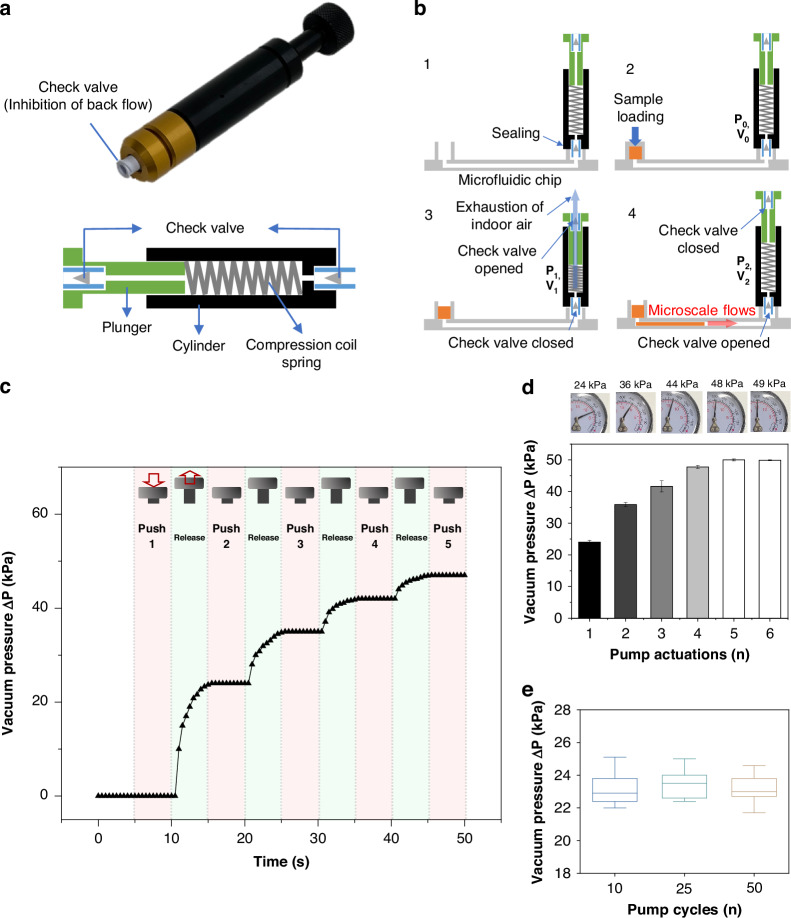
Manual suction pump design and characterization of vacuum generation. **a** Structural components of the pump, including a plunger, compression spring, cylinder, and check valves. **b** Schematic illustration of the operating sequence when connected to the microfluidic chip. **c** Real-time vacuum pressure profile generated by sequential pump actuations. **d** Pressure-time profiles generated by 10 different users (*n* = 10) show very consistent vacuum pressures, regardless of individual pushing forces. **e** Pressure stability over repeated pump cycles

A compact and easy-to-use flow generator is essential for portable biosensor platforms. We therefore developed a manual suction-based pump that operates without electricity. The pump consists of a plunger, push button, compression spring, and two check valves (Fig. 3a). When the button is pressed, air inside the plunger is expelled while the first valve prevents backflow from the chip into the pump. Upon release, the spring restores the button to its original position, generating negative pressure that drives fluid through the chip. The second valve blocks external air from re-entering, maintaining the vacuum and ensuring stable flow (Fig. 3b).

Pumping performance was quantified using a vacuum gauge, and the vacuum level generated during pump operation was recorded as a vacuum–time profile (Fig. 3c). To investigate the potential for flow instability, the pump was intentionally pushed to its end point over a prolonged duration of 5 s. This slow-motion actuation was designed as a rigorous test to observe any fine pulsations that might occur under manual operation. It is noteworthy that even under this extended push motion, no observable pulsation was generated within the microchannel. This stable pressure-driven mechanism enabled steady, unidirectional flow from the inlet to the outlet without any backflow. Such non-pulsatile flow is crucial for maintaining consistent residence times and ensuring reliable nanoparticle interaction within the serpentine mixing stages, regardless of the manual actuation speed. Upon releasing the button to its original position, a negative pressure of approximately 24.0 kPa was consistently generated within 5 s.

We also conducted multiple-push experiments to determine the maximum vacuum capacity of the manual pump. As each successive push was performed only after the push button had returned to its original position, the vacuum pressure gradually accumulated within the chamber. This stepwise progression reached a saturation point of approximately 49.9 kPa after five pushes (Fig. 3c). This observation confirms that the internal check valves effectively maintain the generated vacuum between actuations, ensuring that the final driving pressure is determined by the mechanical properties of the spring rather than the speed or force of individual manual strokes. The generated negative pressure is sufficient to reliably drive fluid through the serpentine microchannel and reaction chamber without the need for an external pump or power source, enabling simple manual operation of the microfluidic system.

To further verify the system’s robustness against inter-user variability, pressure variations were measured across 10 independent users (five males and five females, Fig. 3d). A single push by ten independent users generated a negative pressure of 24.0 kPa with a coefficient of variation of 2.3%, demonstrating the high operational reliability of the manual pumping mechanism. Multiple-push experiments also exhibited consistent stepwise pressure accumulation, confirming that the vacuum buildup is highly reproducible regardless of the specific pressing force applied by each operator user (CV < 4.5% for every push count). For these multiple actuations, the pump was operated in a sequence of successive pushes without prolonged pauses to reach its vacuum saturation point rapidly. While the 5-s prolonged push was used specifically to verify the absence of pulsation, this rapid actuation also ensures that the saturated pressure regime (~49.9 kPa) is established consistently. Notably, the time required to reach the saturated pressure was remarkably uniform, ranging only from 16 to 17 s across all ten users, further confirming that the fluidic initiation is primarily governed by the restoring force of the internal compression spring. The tunable pressure range of our pump allows for operational flexibility. The single-push mode is ideal for streamlined point-of-care testing, while the multiple-push capability offers the potential to drive fluids through more intricate and high-resistance microfluidic networks.

Furthermore, the pump demonstrated excellent mechanical durability by maintaining stable vacuum pressures over repeated operating cycles (10, 25, and 50 cycles), as shown in Fig. 3e. This saturated and stable pressure regime ensures that the assay is performed under a consistent flow environment, minimizing the sensitivity of the assay output to minor flow deviations inherent in manual operation. This repeatable and stable pressure generation indicates that the manual pump can provide consistent flow control, which is paramount for ensuring the reliability and accuracy of practical point-of-care diagnostic applications.

**Fig. 4:**
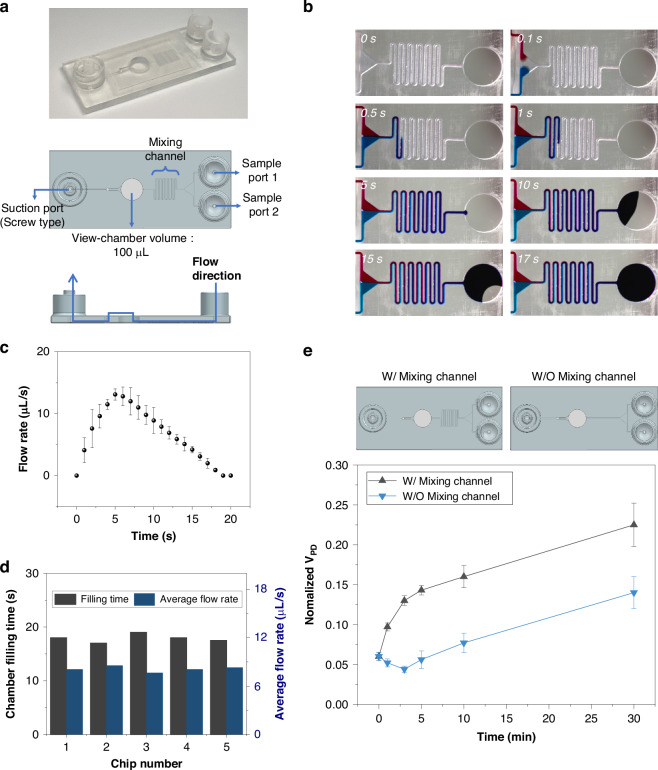
Microfluidic chip design and mixing performance. **a** Chip design with sample ports, suction port, view chamber (100 µL), and serpentine mixing channel. **b** Time-lapse images showing fluid mixing in the channel. **c** Time-dependent flow rate inside the microfluidic chip after a single actuation of the manual suction pump. **d** Chamber filling time and average flow rate across five independent chips (*n* = 5, CV ≈ 4.1%) for evaluation of chip-to-chip reproducibility. **e** Comparison of scattering signals with and without the mixing channel

To demonstrate compatibility, we designed a microfluidic mixing chip tailored for the AuNPs aggregation-based wash-free assay (Fig. 4a). The chip incorporates serpentine channels for reagent mixing, a viewing chamber for scattering measurements, and a Luer-lock screw connector at the outlet for secure attachment to the pump. We established the single-push protocol as the standard for our mixing chip operation, prioritizing user convenience and fluidic stability. While the pump can generate higher pressures, we found that a single actuation provides the most stable and continuous flow, effectively preventing potential flow instabilities or air entrapment that can occur at higher pressure gradients (Fig. S10). Mixing behavior in the serpentine channel was visually demonstrated using red and blue dye solutions (Fig. 4b). Upon a single pump actuation, the liquid rapidly propagated through the channel and achieved complete filling of the view chamber within approximately 20 s. The time-dependent flow rate was further analyzed, showing an average flow rate of approximately 8 µL/s (≈480 µL/min) across five independent chips (Fig. 4c, d). Although the flow rate gradually decreased over time due to the decay of the generated vacuum pressure, the initial high flow enables rapid reagent transport and vigorous mixing within the microchannel. This characteristic is particularly advantageous for time-sensitive biosensing assays, ensuring rapid reaction kinetics. Furthermore, a chip-to-chip variation test using five different chips demonstrated highly reproducible flow generation (CV ≈ 4.1%), confirming the robustness of the integrated platform (Fig. 4d).

The integrated pump–chip system was applied to the thrombin assay to evaluate the influence of microfluidic design on sensor performance (Fig. 4e and Video S2). TBA-functionalized AuNPs and NaCl solution were introduced into separate inlets, with thrombin (16 nM) added to the AuNP suspension. To quantify the benefit of the serpentine geometry, we compared the serpentine channel with a conventional straight channel under identical pumping conditions. The serpentine channel induced secondary Dean vortices, facilitating chaotic advection that significantly accelerated the mixing of ions and reagents^55,56^. Notably, the serpentine design achieved V_PD_ signals nearly twice as high as those of the straight channel (Fig. 4e). We attribute this enhancement to the initial kinetic advantage provided by rapid mixing during the early stage of the assay. In the serpentine channel, the vigorous Dean flow effectively reduces the diffusion barrier, ensuring that the NaCl-mediated electrostatic screening occurs uniformly and simultaneously across the entire nanoparticle population. This rapid initial homogenization promotes the formation of a higher density of aggregation ‘nuclei’ or seeds. In colloidal systems, these early-stage aggregation events dictate the subsequent growth and coupling kinetics over the entire 30 min incubation period. In contrast, the straight channel relies on slow molecular diffusion, leading to localized and incomplete reaction bottlenecks that cannot be fully compensated for even with extended time. These results demonstrate that serpentine architecture is essential for maximizing the aggregation-induced scattering signal, confirming that the initial mixing efficiency is the primary determinant of the final sensing performance. Together, these findings validate that our integrated pump–chip platform delivers stable, user-independent flow control and rapid mixing without external power, establishing a robust and scalable framework for portable point-of-care diagnostics.

### Protein assays using portable integrated optical biosensor platform

Finally, we evaluated the clinical applicability of the portable integrated optical biosensor by detecting two representative protein biomarkers, thrombin and the SARS-CoV-2 spike protein. For thrombin detection, solutions of varying concentrations (0.25–16 nM) were injected into the microfluidic device containing TBA-functionalized AuNPs, and 0.1 M NaCl was supplied through the second inlet and mixed using a hand pump. The scattering signal was recorded for more than 5 min, and the normalized response was defined as the ratio of the final photodiode voltage to the initial photodiode voltage.

**Fig. 5:**
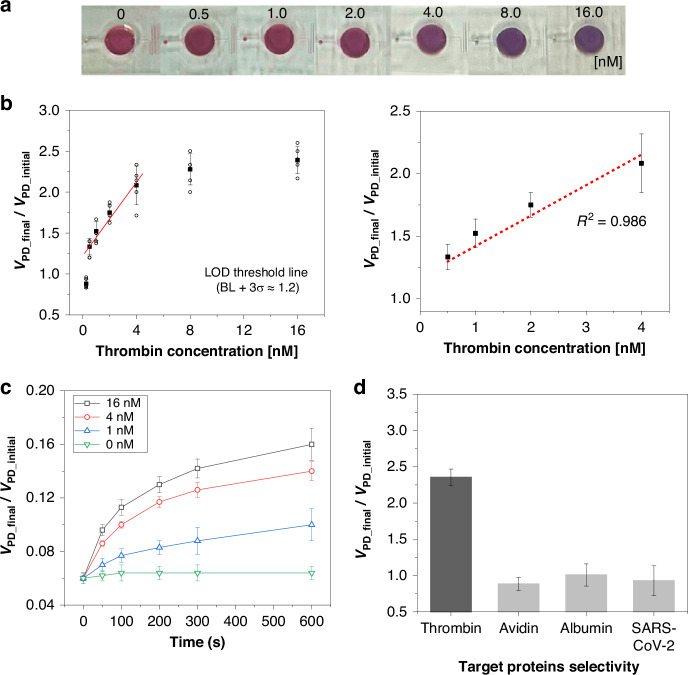
Thrombin detection using the portable microfluidic platform in 1× PBS buffer. **a** Colorimetric change of AuNPs with increasing thrombin concentrations. **b** Normalized scattering signals increase proportionally with thrombin concentration (0.25–16 nM), showing a linear calibration curve (R² = 0.986, *n* = 5, error bars = SD). **c** Real-time V_PD_ monitoring at different concentrations. **d** Selectivity test against non-target proteins

Thrombin-induced aggregation was clearly visualized by a plasmon color change from red to purple (Fig. 5a). The normalized scattering signal increased proportionally with thrombin concentration (Fig. 5b) and showed a linear response in the range of 0.5–4 nM before reaching saturation (R² = 0.986). The statistical limit of detection (LoD, 3σ from baseline) was determined to be 0.5 nM, which is lower than clinically relevant thrombin concentrations (10–30 nM)^42,45^. This sensitivity indicates that the platform can detect thrombin at physiologically meaningful levels while maintaining a rapid and wash-free assay format. Real-time monitoring confirmed concentration-dependent aggregation kinetics (Fig. 5c). Selectivity tests for non-target proteins, including avidin, human serum albumin (HSA), and the SARS-CoV-2 spike protein, showed minimal responses (Fig. 5d), confirming the specificity of the aptamer-AuNPs assay.

**Fig. 6:**
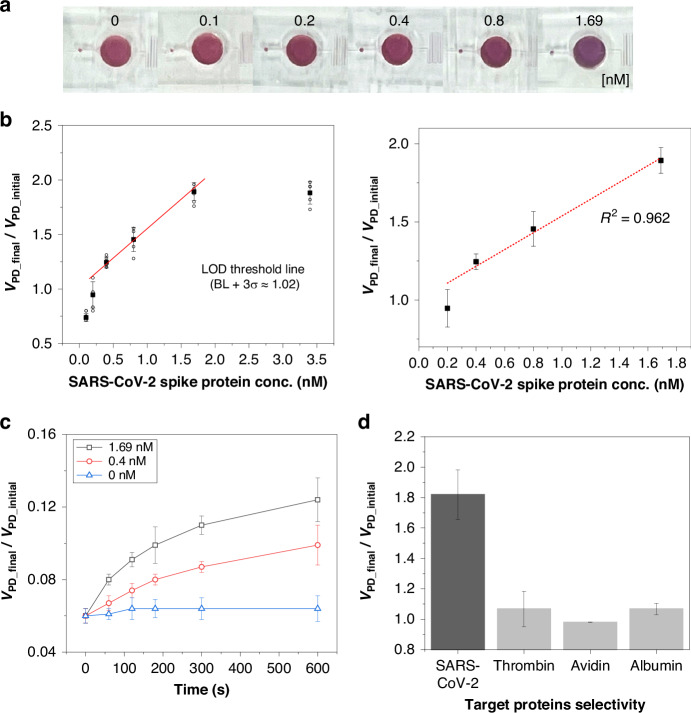
Detection of SARS-CoV-2 spike protein using the portable microfluidic platform in 1× PBS buffer. **a** Colorimetric change of AuNPs with increasing spike protein concentrations. **b** Normalized scattering signals increase proportionally with concentration (0.1–3.4 nM), showing a linear calibration curve (R² = 0.962, *n* = 5, error bars = SD). **c** Real-time V_PD_ monitoring at different concentrations. **d** Selectivity test against non-target proteins

To further demonstrate the versatility of the assay, the platform was applied to detect the SARS-CoV-2 spike protein under the same conditions. Distinct color and scattering changes were observed in the presence of the spike protein (Fig. 6a, b). The normalized scattering signal increased linearly with concentration from 0.2 nM to 1.69 nM (R² = 0.962, Fig. 6c), and the LoD was calculated to be 0.2 nM. As with thrombin, the photodiode signal tracked the aggregation reaction rate in real time (Fig. 6c). Selectivity analysis revealed negligible cross-reactivity with thrombin, avidin, and HSA (Fig. 6d).

These results demonstrate that the proposed sensing strategy is not limited to a single biomarker but can be readily extended to other protein targets by simply replacing the aptamer sequence. Since the sensing principle relies on target-induced aggregation of AuNPs combined with optical scattering detection, the platform provides a highly flexible and modular approach for rapid biomarker detection. Furthermore, the intrinsic modularity of the device design allows for potential multiplexing through the integration of parallel microfluidic channels, enabling the simultaneous screening of multiple pathogens in a single operational step. Collectively, these findings suggest that the integrated biosensor has the potential to sensitively and selectively identify multiple protein biomarkers within a simplified, wash-free workflow.

### Protein assay performance in complex biological matrices

To further assess clinical applicability, the assay was tested in complex biological matrices. First, the effect of human serum albumin (HSA) was examined (Fig. S11). Increasing HSA concentrations (0–1000 µM) progressively reduced the normalized scattering intensity, consistent with partial inhibition of AuNPs aggregation and potential interference with thrombin-aptamer interactions. The relative intensity signal decreased from approximately 2.5 in the absence of HSA to ~1.45 at 200 µM and approached the baseline level at higher concentrations. Notably, the signal at 200 µM remained above the limit-of-detection threshold (BL + 3σ ≈ 1.3), indicating that thrombin detection is still achievable at moderate albumin concentrations. However, at 500 µM, comparable to physiological serum levels (530–760 µM)^57^, the signal approached the detection threshold, suggesting that abundant serum proteins can significantly attenuate nanoparticle aggregation signals. This limitation may be mitigated in future work by incorporating rapid pretreatment strategies, such as commercial albumin depletion kits^58^.

Spike-in validation was then performed using thrombin in synthetic urine and SARS-CoV-2 spike protein in synthetic saliva (Figs. S12, S13). This design reflects clinically relevant scenarios, as urinary thrombin has been reported as a marker of renal and glomerular pathology, while salivary SARS-CoV-2 spike protein is a well-established non-invasive biomarker for viral infection^43–46^. In synthetic urine, increased ionic strength promoted AuNPs aggregation compared with PBS, resulting in a higher baseline signal even in the absence of thrombin. Although this value exceeded the buffer-based LoD, it remained within concentration ranges reported in previous urine-based thrombin assays^59^. This demonstrates the feasibility of our portable platform for thrombin detection in complex biological matrices. In contrast, in synthetic saliva, higher viscosity retarded particle aggregation, leading to weakened colorimetric contrast and reduced scattering intensity. The LoD for the SARS-CoV-2 spike protein in saliva was determined to be 0.8 nM, representing an improvement over previously reported optical aptasensors that exhibited detection limits in the tens of nanomolar range^60^. Although matrix effects limited the magnitude of the signal change, the assay maintained sensitivity in both urine and saliva, underscoring its robustness toward clinically relevant fluids.

This study, however, focused on validating the platform using synthetic matrices to establish a robust performance baseline while minimizing unpredictable biological variables. While synthetic models are essential for initial proof-of-concept and device optimization, further validation using actual patient-derived samples is necessary to fully evaluate diagnostic performance in complex clinical settings. This study, therefore, serves as a pivotal intermediate step toward full-scale clinical translation.

### Comparative analysis and practical advantages

To highlight the practical advantages of our integrated platform, we performed a comprehensive comparison with conventional diagnostic methods and previously reported aptasensor (Table S1). Unlike standard Sandwich ELISA or RT-PCR, which typically require 1.5 to 4 h of assay time and multiple laborious washing steps (≥2–3 steps), our plasmonic scattering platform achieves target detection within only 5 min with zero washing steps. Furthermore, while lateral flow assays (LFAs) offer comparable simplicity and speed, they often suffer from poor reproducibility and semi-quantitative results due to user-dependent interpretations and inconsistent flow rates. In contrast, our integrated system ensures high quantitative precision and user-independent reproducibility by standardizing the fluidic initiation through a manual, spring-driven suction pump. This drastic reduction in complexity and time is achieved by the “turn-on” nature of the scattering signal and the efficient mixing provided by the handheld pump and serpentine microchannel.

Furthermore, our platform demonstrates superior sensitivity compared to several state-of-the-art optical biosensors (Table S2)^60–67^. For thrombin detection, the achieved LoD of 0.5 nM is lower than that of various Surface Plasmon Resonance (SPR) and optical fiber-based sensors, which report LoDs ranging from 0.9 nM to 36 nM. Similarly, for the SARS-CoV-2 spike protein, our platform attained a LoD of 0.2 nM in buffer, significantly outperforming D-shaped plastic optical fiber aptasensors that report an LoD of approximately 36.7 nM^62^. It is also noteworthy that the analytical sensitivity in complex biological fluids, specifically 4 nM in synthetic urine and 0.8 nM in synthetic saliva, remains highly competitive despite the signal attenuation caused by matrix effects. By bridging the gap between sophisticated laboratory analysis and portable, user-independent operation, our platform establishes itself as a robust and scalable alternative for next-generation point-of-care diagnostics.

## Conclusions

In this study, we developed a fully integrated and portable microfluidic biosensor platform that enables rapid, wash-free bioassays utilizing aptamer-mediated AuNPs aggregation. The platform successfully integrates three key innovations: a miniaturized optical detection system, an electricity-free manual suction pump driven by a compression spring, and a disposable microfluidic mixing chip. A significant advancement of this system is its ability to ensure user-independent reproducibility (CV < 4.5%) while promoting Dean flow-induced inertial mixing within the serpentine microchannels, which accelerates aggregation kinetics without external power. Compared with conventional biosensing systems that require bulky instruments or multi-step protocols, our platform provides a self-contained solution for detecting clinically relevant biomarkers, including thrombin and the SARS-CoV-2 spike protein, within only 5 min. The feasibility of the platform was further validated through spike-in assays in complex biological matrices such as synthetic urine and saliva, maintaining sub-nanomolar sensitivity. The device achieves palm-sized portability while the low-cost, 3D-printed disposable chip minimizes cross-contamination. By combining electricity-free flow control, real-time plasmonic signal measurement, and wireless communication, this platform establishes a robust and field-deployable approach for rapid, decentralized diagnostic applications.

## Supplementary information

Supporting Information

Supplementary Video S1

Supplementary Video S2
