# Supplementary material for: Microfluidic–optical integrated portable platform with handheld pump for wash-free plasmonic detection of thrombin and SARS-CoV-2 spike protein
Source: Microsyst Nanoeng. 2026 Jul 17;12:266. doi: 10.1038/s41378-026-01381-3 (PMC13379383; doi:10.1038/s41378-026-01381-3)
Supplement: Supplementary file 1 — Supporting Information [file 41378_2026_1381_MOESM1_ESM.docx]

Supporting Information

Microfluidic–Optical Integrated Portable Platform with Handheld Pump for Wash-Free Plasmonic Detection of Thrombin and SARS-CoV-2 Spike Protein

Da-In Kwon^ab^, Yeong-Eun Yoo^a^ Jae-Ho Jin^c^, Ji Hyo Park^a^, Ga Eun Han^a^, Kwanoh Kim^a^, Jae Sung Yoon^a^, Seong Min Kang^b^* and Do Hyun Kang^ade^*

^a^ Nano-Lithography & Manufacturing Research Center, Korea Institute of Machinery and Materials (KIMM), Daejeon 34103, South Korea.

^b^ Department of Mechanical Engineering, Sogang University, Seoul 04107, South Korea

^c^ Neo Nanotech Co., Ltd., Daejeon 34103, South Korea.

^d^ Department of Chemistry, Chung-Ang University, Seoul 06974, South Korea

^e^ Advanced Bioconvergence, University of Science & Technology, Daejeon 34113, South Korea.

CORRESPONDING AUTHOR EMAIL ADDRESS:

[smkang@sogang.ac.kr](mailto:smkang@sogang.ac.kr) and [dhkang@kimm.re.kr](mailto:dhkang@kimm.re.kr)

**Scheme S1. Schematic representation of the synthesis and functionalization of Au NPs with aptamers.** Gold ions (Au³⁺) were reduced by trisodium citrate at 100 ℃ for 20 minutes, forming citrate-stabilized gold nanoparticles (AuNP-Citrate) through the classical Turkevich method. Aptamers (ssDNA) are introduced to displace the surface-bound citrate molecules on AuNPs, resulting in the formation of AuNP-aptamer conjugates. Upon target binding, the aptamers exhibit strong affinity for thrombin and are desorbed from the nanoparticle surface, leaving the Au NPs bare.


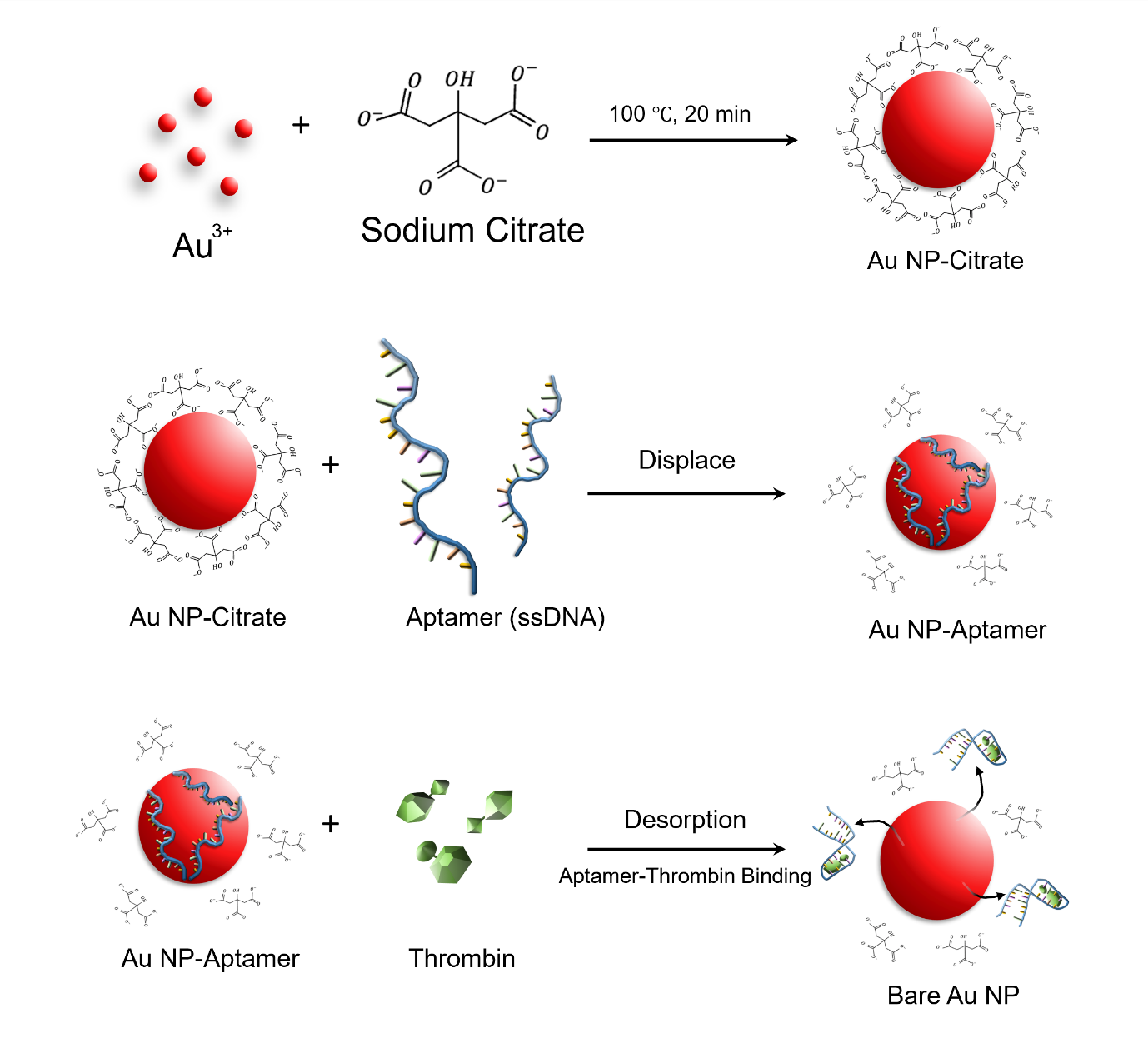


**Figure S1. Characterization of citrate-stabilized Au NPs. A)** UV-vis spectra of AuNPs synthesized in three independent batches (n = 3). Spectra were normalized to their respective maximum absorbance values. The SPR peak positions were 518.6, 519.0, and 518.7 nm (mean ± SD = 518.8 ± 0.2 nm), demonstrating excellent batch-to-batch consistency. The insect shows a photograph of the synthesized AuNPs solution. **B)** DLS analysis shows an average hydrodynamic diameter of 24.3 nm (n = 3; ± 0.38 nm SD, ± 0.22 nm SE, 95% CI ± 0.93 nm). **C)** Zeta potential is −35 mV, indicating good colloidal stability (n = 3; ± 3.14 mV SD, ± 1.81 mV SE, 95% CI ± 7.81 mV).

*SD: Standard Deviation, SE: Standard Error, CI: Confidence Interval.*


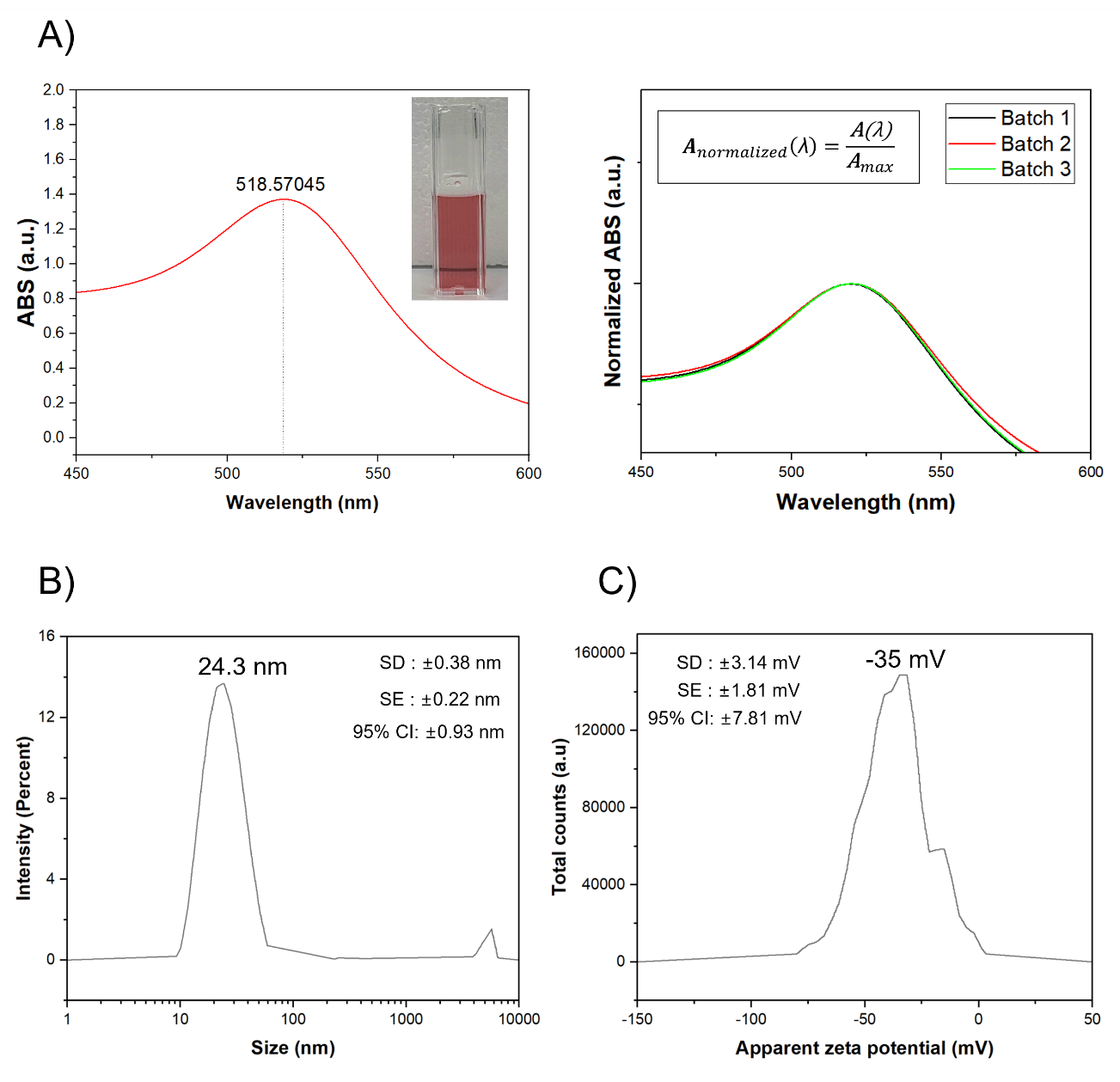


**Figure S2. Colloidal stability of citrate-capped AuNPs stored at 4 ℃ over 180 days.** The plasmonic peak remained at 518 nm for up to 7 days, with a slight red shift to 520 nm observed on day 180, indicating excellent long-term stability.


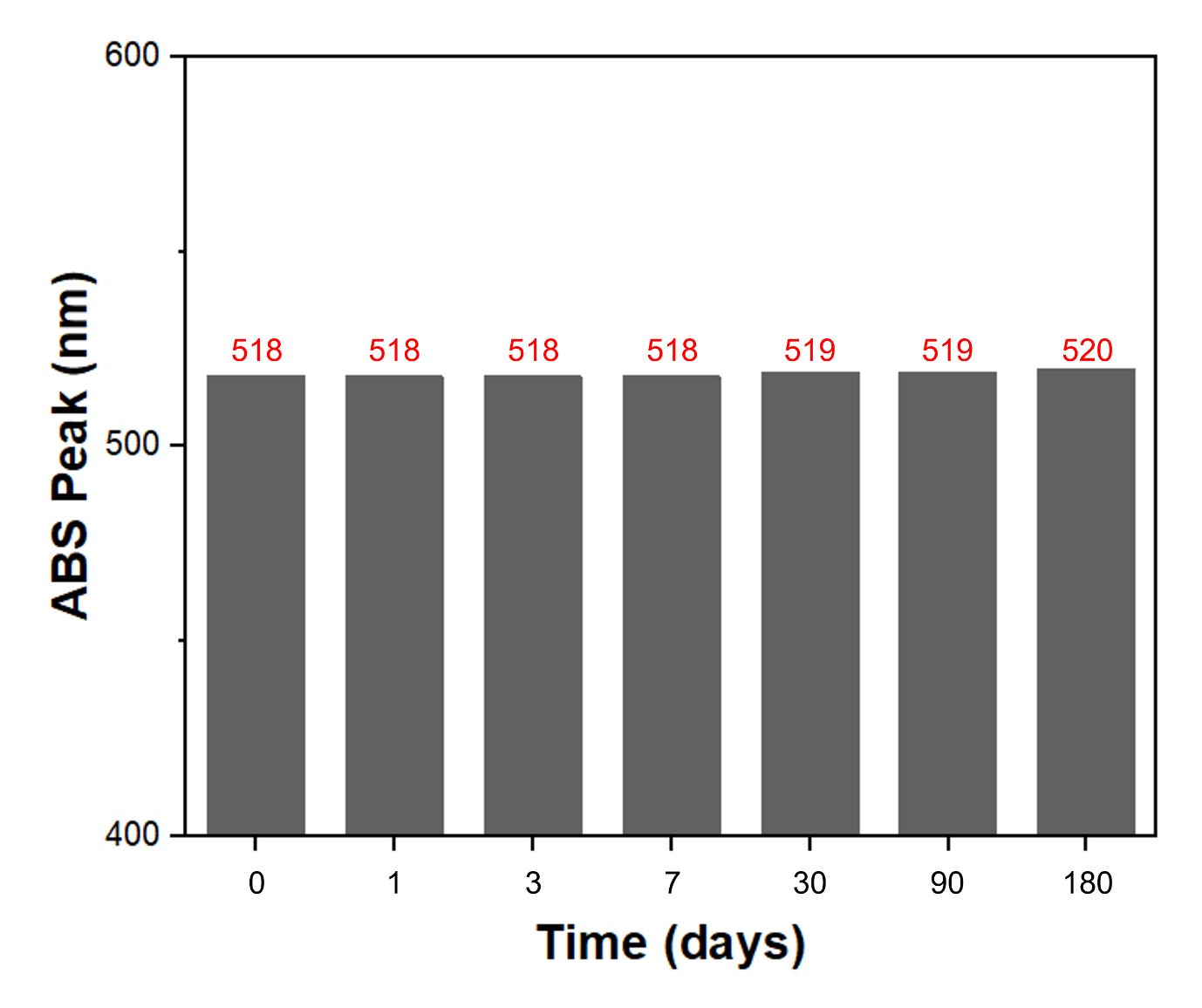


**Figure S3. Experimental setup for plasmonic scattering measurements using a spectrometer.** A 638 nm laser source was directed onto the sample chamber (100 µL coverwell on a glass substrate), and the scattered light was collected at a 45° angle by an optical fiber connected to a spectrometer. The measurements were performed under a dark enclosure to minimize background interference, enabling real-time monitoring of AuNP aggregation. This spectrometer-based configuration was employed to conduct prior optimization experiments before applying the assay to the portable platform.


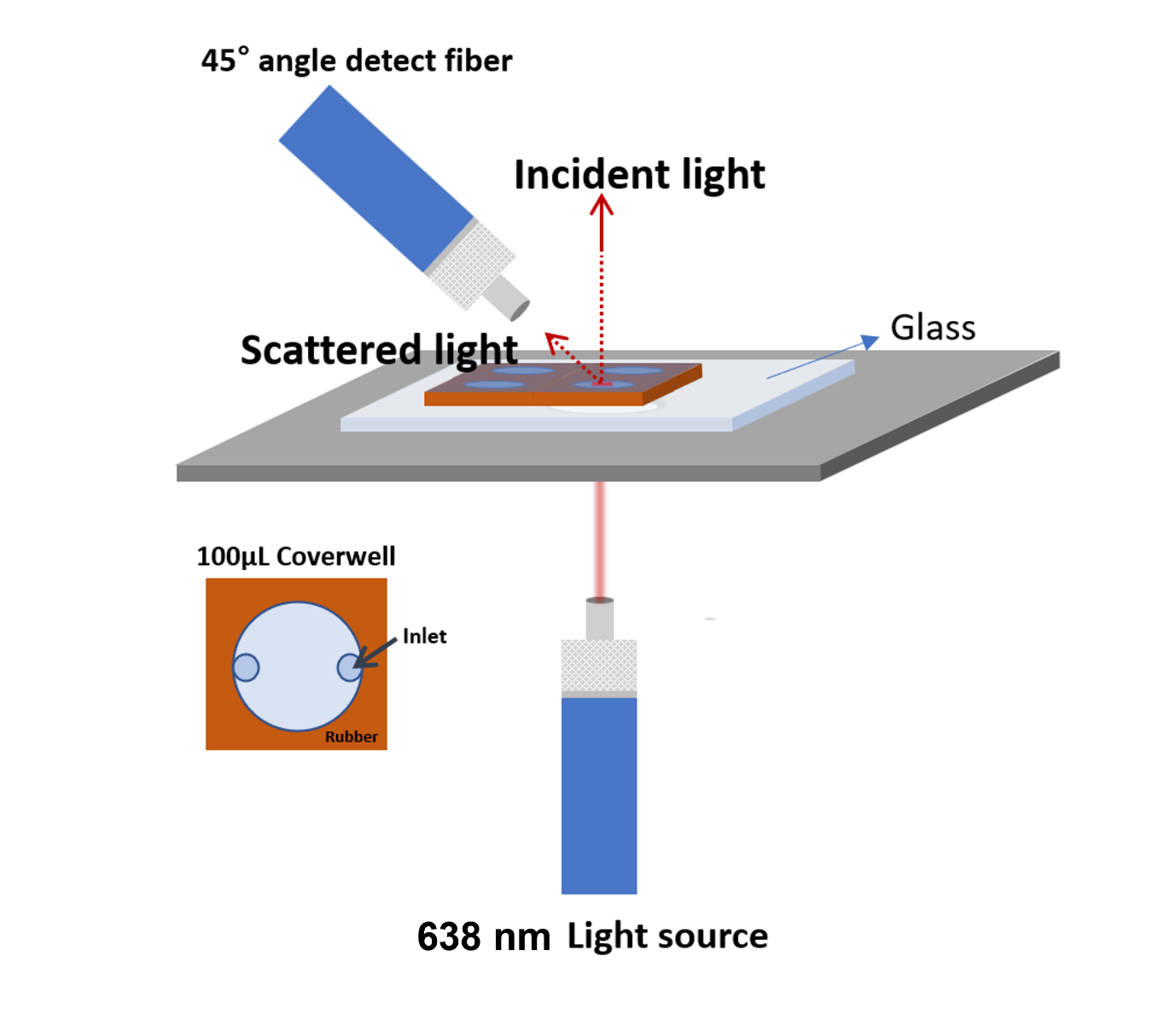


**Figure S4. Optimization of aptamer concentration for thrombin detection. A)** The graph shows the scattering intensity of TBA-functionalized Au NPs at varying aptamer (TBA) concentrations (0–10 µM) after inducing aggregation with 0.1 M NaCl. **B)** The image visually compares nanoparticle solutions at different TBA concentrations, illustrating a color change from red to a more dispersed state as the TBA concentration increases.


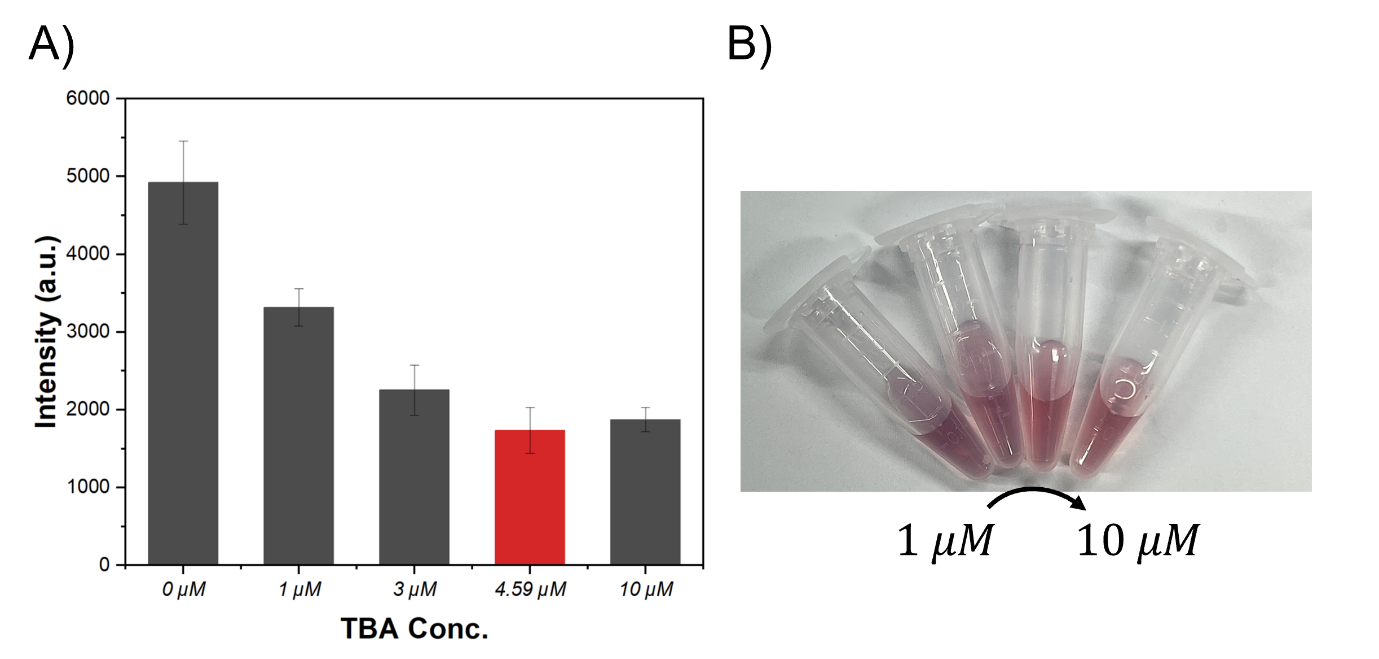


**Figure S5. Optimization of NaCl concentration for aggregation-based detection.** **A)** The graph shows the scattering intensity of TBA-functionalized Au NPs (4.59 µM) in the presence of different NaCl concentrations (0.1 M, 0.5 M, and 1.0 M) for both control and target samples. **B)** Aggregation ratio calculated by dividing the scattering intensity of the target sample by that of the control.


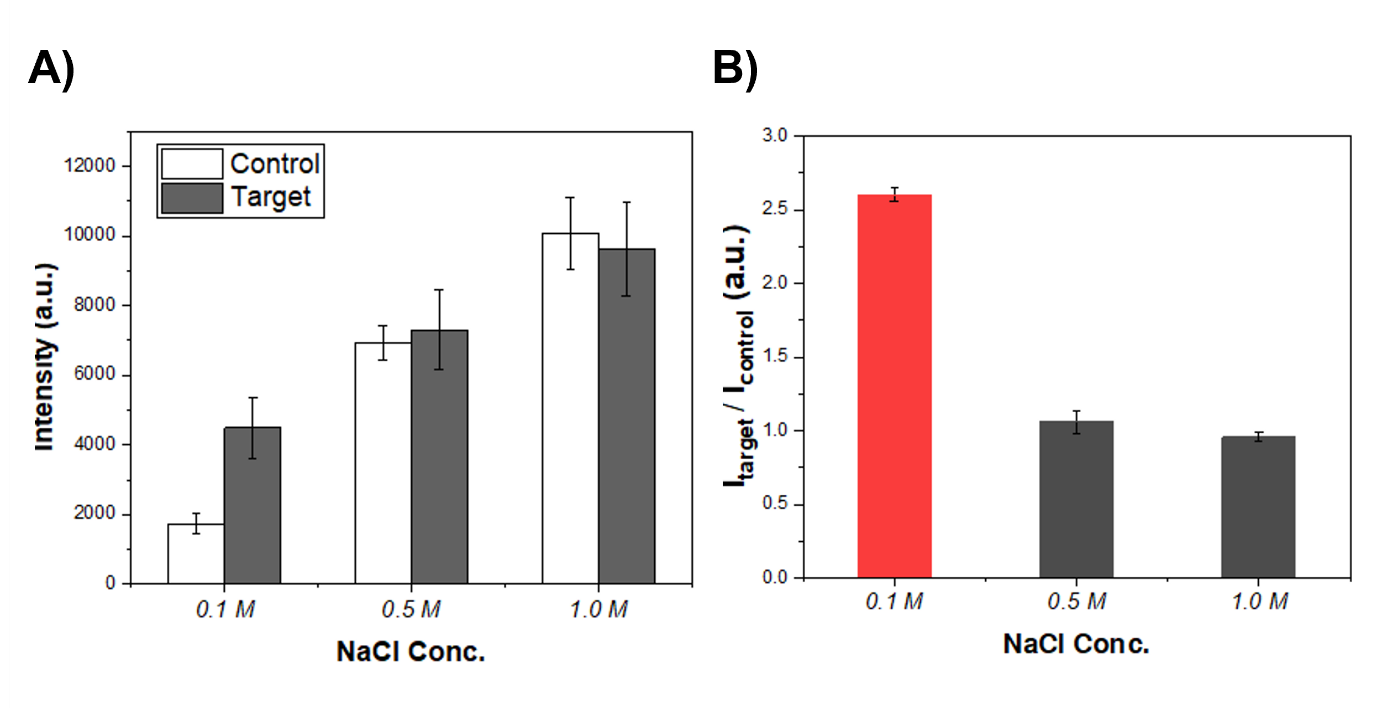


**Figure S6. Effect of buffer pH on thrombin-induced AuNP aggregation.** **A)** UV–vis spectra of AuNP solutions with thrombin (276 nM) in MES (pH 5.7), PBS (pH 7.4), and Tris (pH 8.7) buffers using a calibrated pH meter. **B)** Aggregation ratio (A620 / A520) was highest at pH 7.4.

**
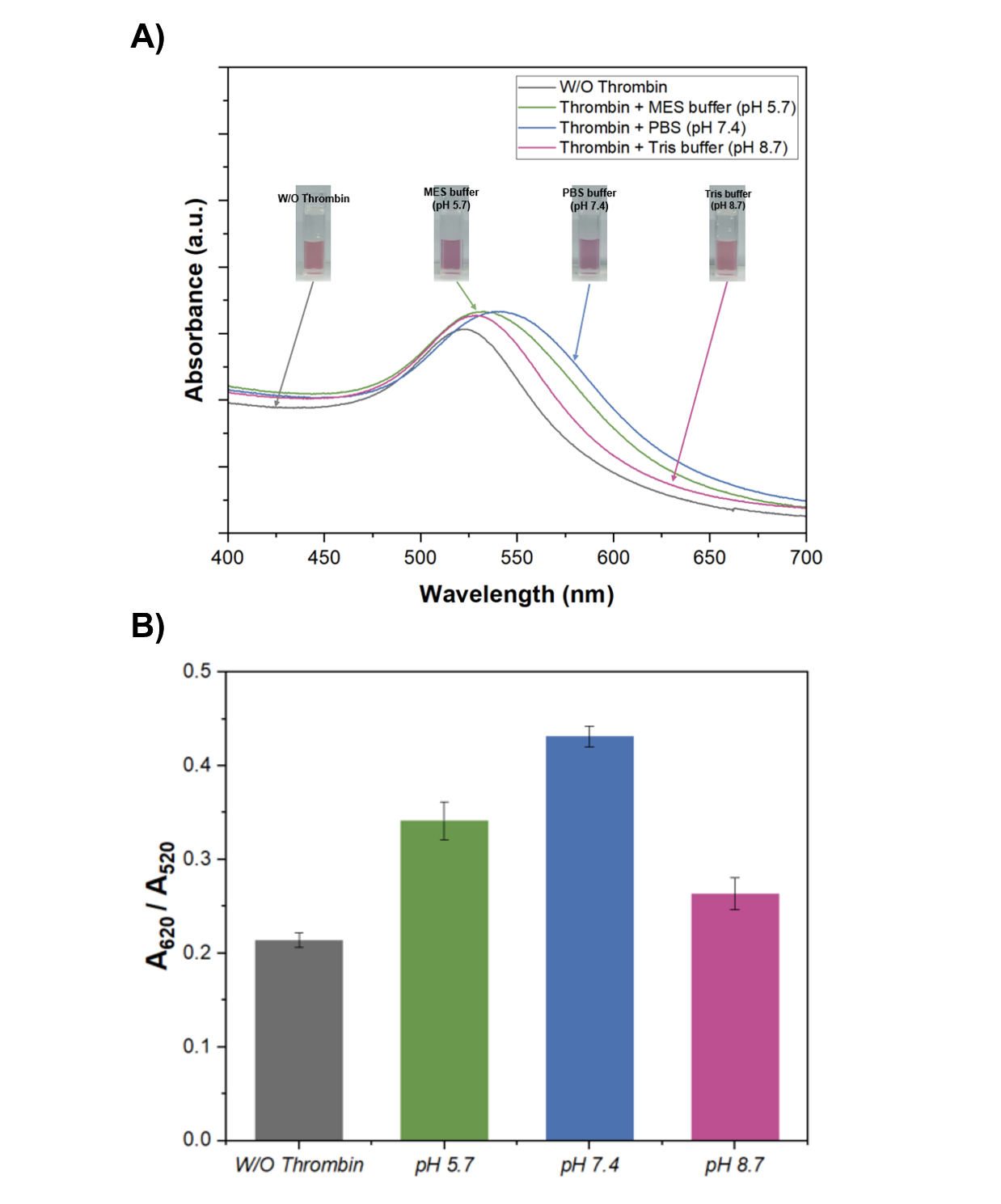
**

**Figure S7. Photograph of the PCB used for wireless sensor operation.** The board integrates a MCU (CC254X), BLE module, power switch, DC-DC converter, and a 3.7 V, 500 mAh Li-Po battery for signal transmission and device control.


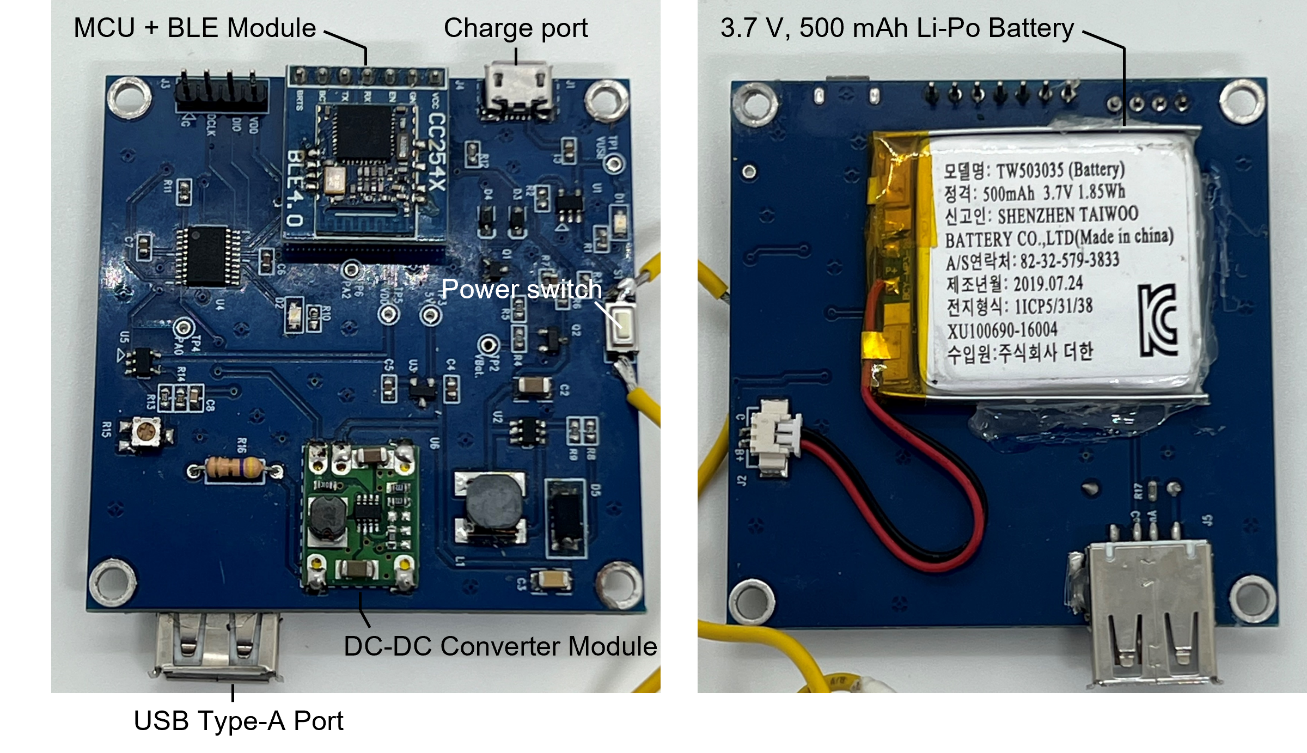


**Figure S8. Schematic illustration of microfluidic chip fabrication.** A 3D-printed microfluidic chip is assembled with an adhesive polyethylene (PE) film (0.15 T) to form a sealed structure. The sealing process ensures proper integration of the components, enabling controlled fluid flow within the chip.

**
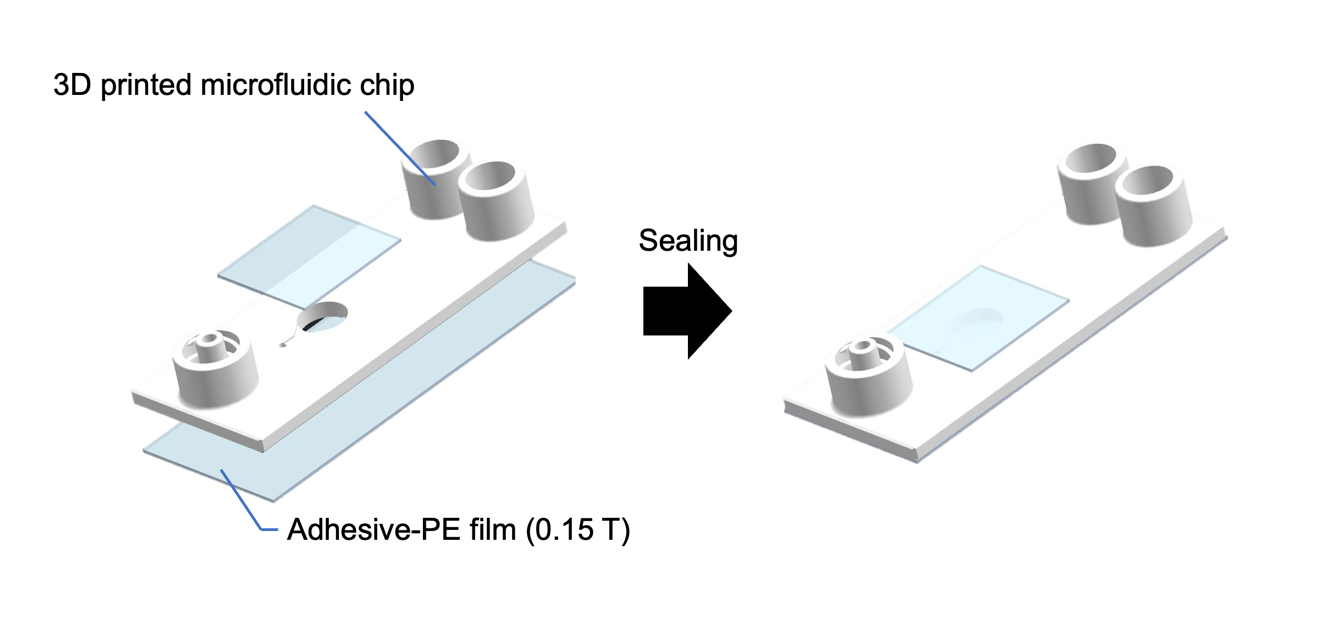
**

**Figure. S9 Effect of photodetector (PD) angle on signal measurement.** The ratio of VPD_final / VPD_initial was measured at different PD angles to evaluate the detection efficiency. The signal intensity increased as the PD angle increased, reaching its highest value at **61.5 degrees**, indicating that this angle provides the most efficient detection condition.


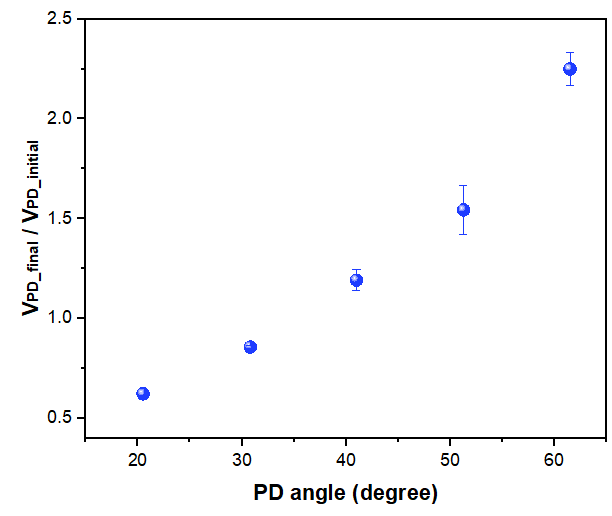


**Figure S10. Effect of pump actuation on chamber filling behavior.** Time-lapse images show chamber filling after two pump actuations. The higher vacuum pressure increases the flow rate, resulting in uneven chamber filling.


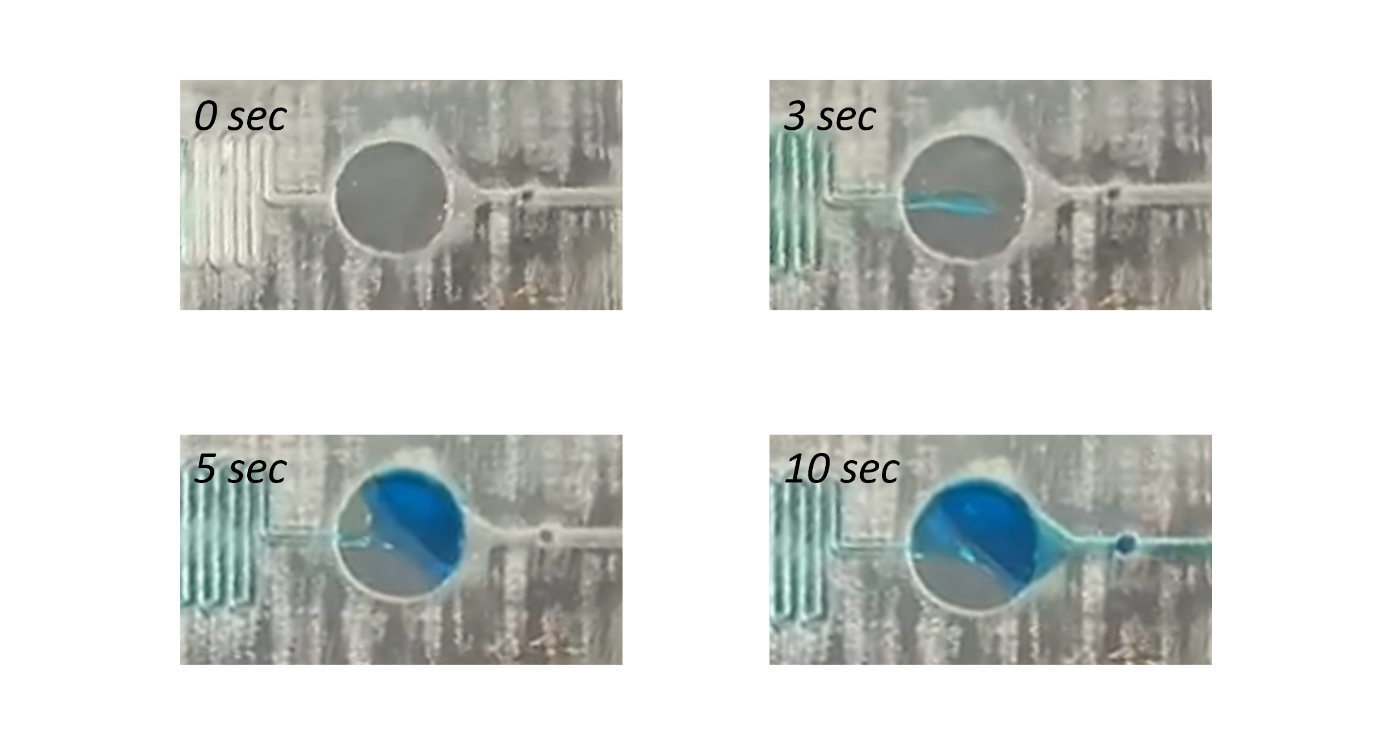


**Figure S11. Effect of human serum albumin (HSA) on thrombin-induced AuNP aggregation.** Scattering intensity ratios (Itarget / Icontrol) of TBA-AuNPs were measured with 16 nM thrombin and 4.59 µM aptamer at varying HSA concentrations (0–1000 µM). Increasing HSA reduced aggregation, but detectable signals were retained at physiological levels (500 µM, n = 3).


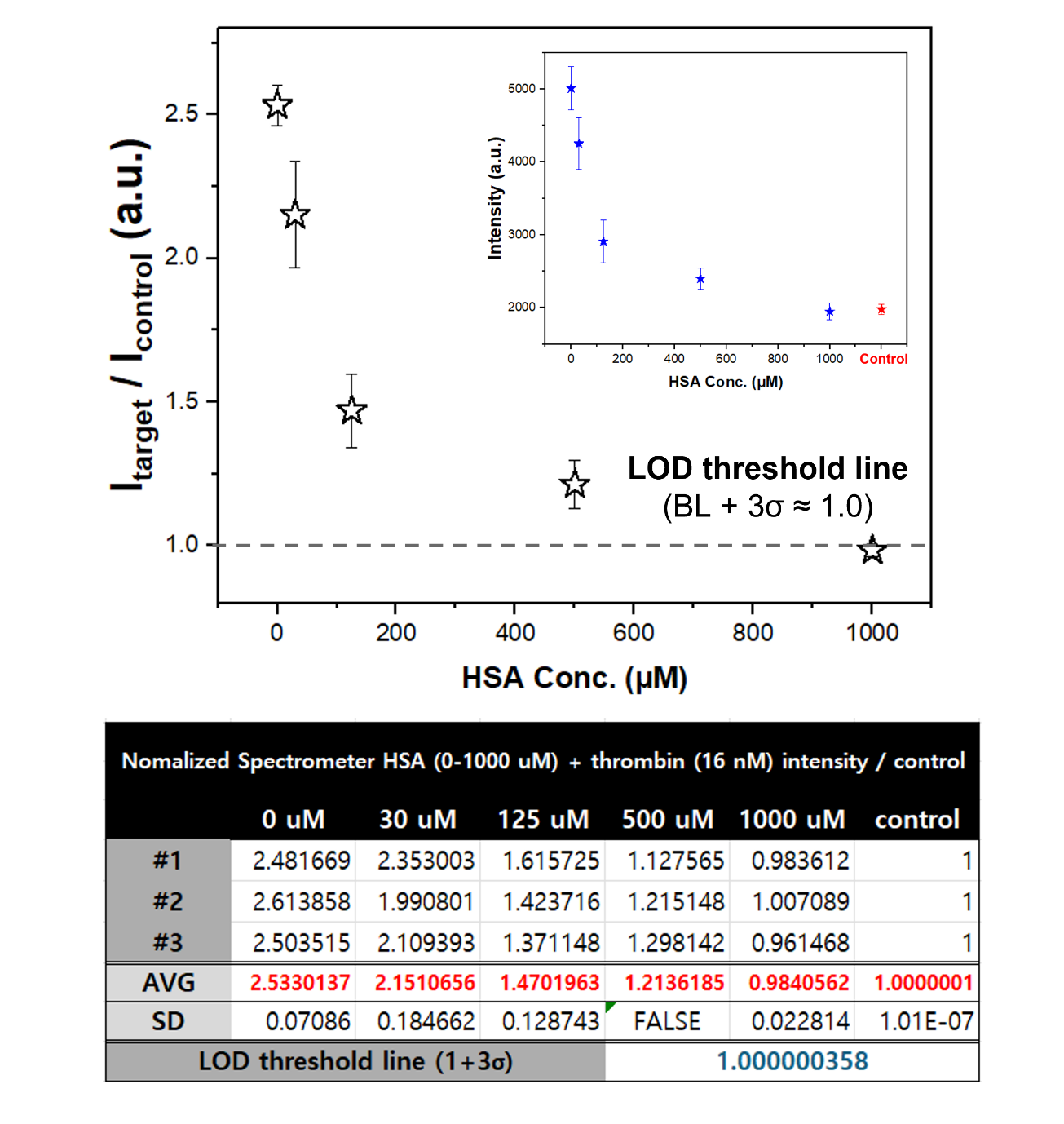


**Figure S12. Detection of thrombin spiked into synthetic urine using the portable biosensor platform.** Normalized scattering signals (VPD_final / VPD_initial) were measured for thrombin (0.1–16 nM) in undiluted synthetic urine. The LoD threshold line was 1.26 (BL + 3σ), and the practical LoD was 4 nM, confirming reliable detection in urine samples (n = 3).


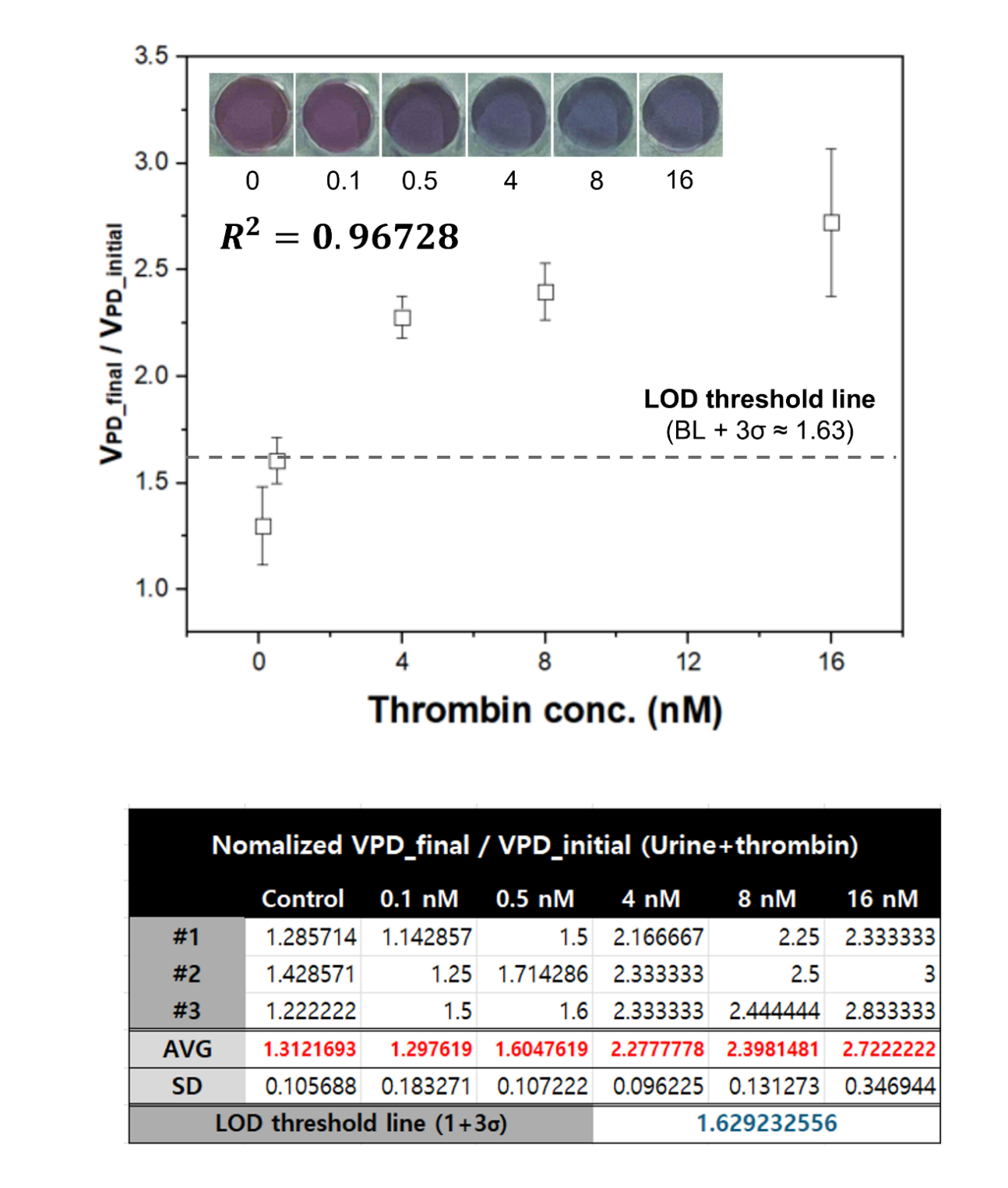


**Figure S13. Detection of SARS-CoV-2 spike protein spiked into synthetic saliva using the portable biosensor platform.** Normalized scattering signals (VPD_final / VPD_initial) were measured for spike protein (0.1–1.69 nM) in synthetic saliva. The LoD threshold was 1.26 (BL + 3σ), and the practical LoD was 0.8 nM (n = 3).


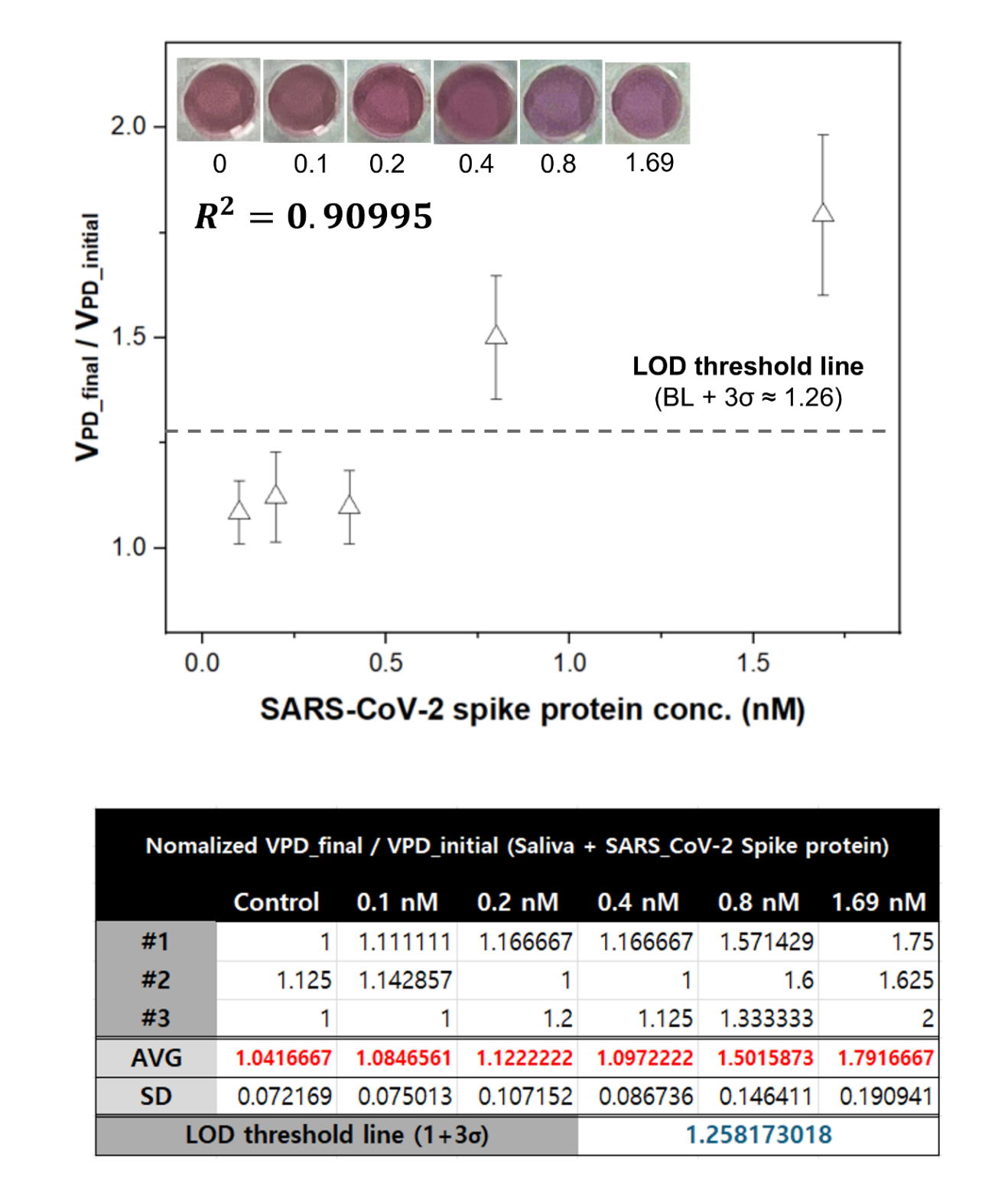


**Table S1.** Comparison of conventional diagnostic methods and aptamer-based sensing platforms for biomarker detection.

| **Sensing Technology** | **Biomolecule** | **Assay time** | **Number of steps** | **Washing** | **LoD** | **Equipment requirement** | **Ref** |
| --- | --- | --- | --- | --- | --- | --- | --- |
| Sandwich ELISA  (ThermoFisher) | SARS-CoV-2 spike | 1.5–3 h | ≥3 | Required | pM–nM | Microplate reader | ^1^ |
| Sandwich ELISA  (ThermoFisher) | Thrombin | 3–4 h | ≥3 | Required |  |  | ^2^ |
| RT-PCR | SARS-CoV-2 RNA | 1.5–2 h | ≥2  (RNA extraction) | Required | 0.2–1.0  Copies/μL | Thermal cycler | ^3^ |
| Conventional  RT-PCR | SARS-CoV-2 RNA | 2–3 h | ≥2  (RNA extraction) | Required |  |  | ^4^ |
| LFA | SARS-CoV-2 antigen | 10–20 min | 1 | None | Qualitative | None | ^5^ |
| Electrochemical  aptamer sensor | Thrombin | 30–60 min | ≥2 | Required | pM | Bench-top SPR instrument | ^6^ |
| Plasmonic Scattering | Thrombin/  SARS-CoV-2 spike | 5 min | 1 | None | 0.4–0.5 nM | Compact optical module | This study |

**Table S2.** Comparison of different optical sensors using aptamer

| **Sensing Technology** | **Biomolecule** | **Labelling** | **Detection type** | **Assay time** | **Dynamic range** | **LoD** | **Ref** |
| --- | --- | --- | --- | --- | --- | --- | --- |
| SPR & Optical Fiber | Thrombin | Label-free | Single Aptamer | 5–10 min | 1.6–60 nM | 1 nM |  |
| SPR | Thrombin | Label-free | Single Aptamer | 15 min | 1–20 nM | 1.1 nM | ^7^ |
| SPR | Thrombin | Label-free | Single Aptamer | 30–60 min | 5–500 nM | 0.9 nM | ^8^ |
| SPR | Thrombin | Label-free | Single Aptamer | 4 min | 1.35–27 nM | 1.35 nM | ^9^ |
| SPR & Optical Fiber | Thrombin | Label-free | Single Aptamer | 60 min- | 100 nM–2 μM | 36 nM | ^10^ |
| Fluorescence | Thrombin | Labeled | Split Aptamer | 30 min | 0–50 nM | 2 nM | ^11^ |
| SPR & Optical Fiber | SARS-CoV-2 spike | Label-free | Single Aptamer | 20 min | 25–1000 nM | 36.7 nM | ^12^ |
| Plasmonic Scattering | Thrombin | Label-free | Single Aptamer | 5 min | 0.5–16.5 nM | 0.5 nM | This study |
| Plasmonic Scattering | SARS-CoV-2 spike | Label-free | Single Aptamer | 5 min | 0.1–1.69 nM | 0.4 nM | This study |

**Supplementary Videos**

**Supplementary Video S1.** Demonstration of device startup, Bluetooth connectivity, and real-time VPD signal display on a smartphone application.


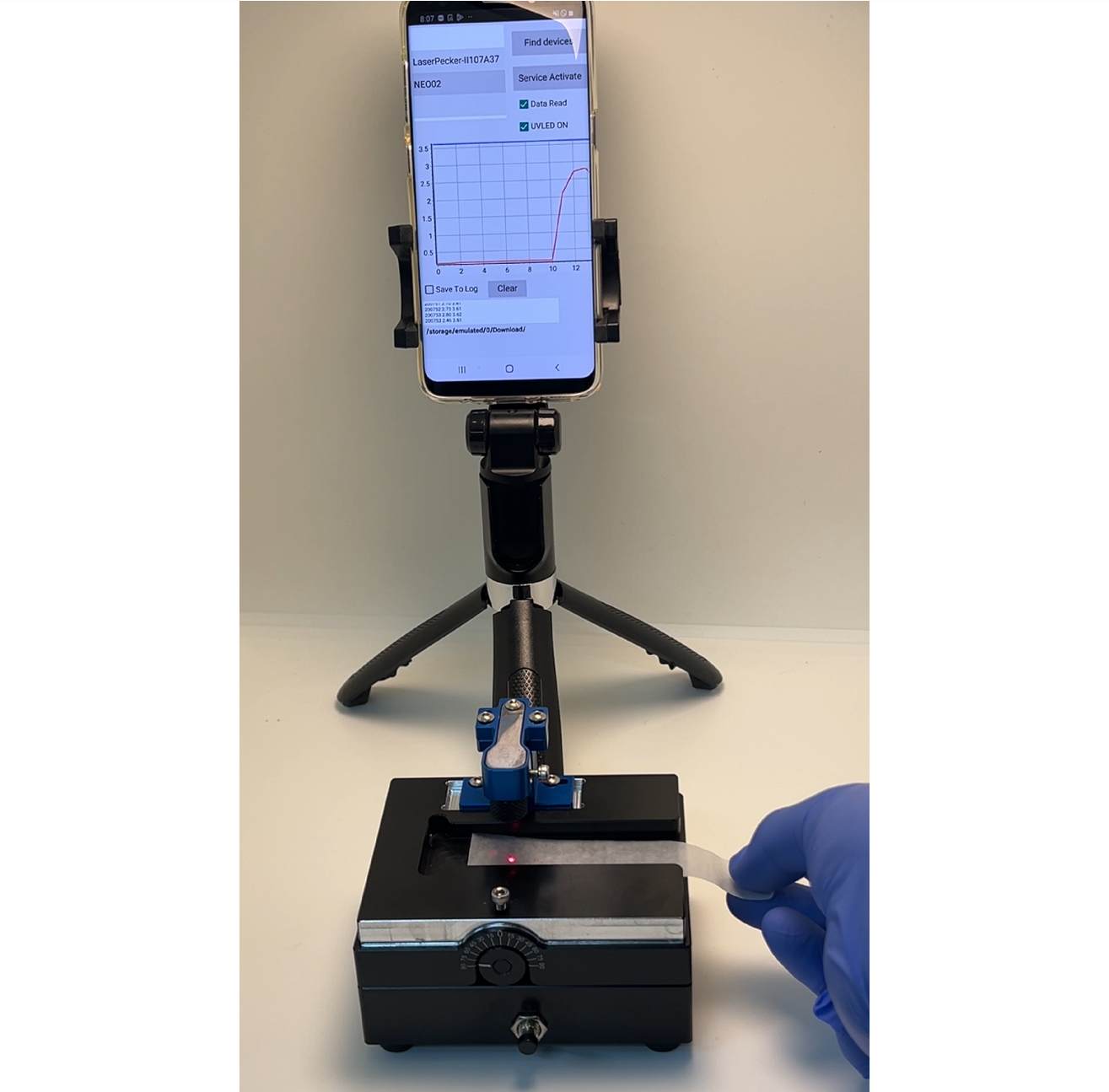


**Supplementary Video S2.** Demonstration of sample loading, chip mounting, and pump actuation for initiating fluid flow in the portable biosensor platform.


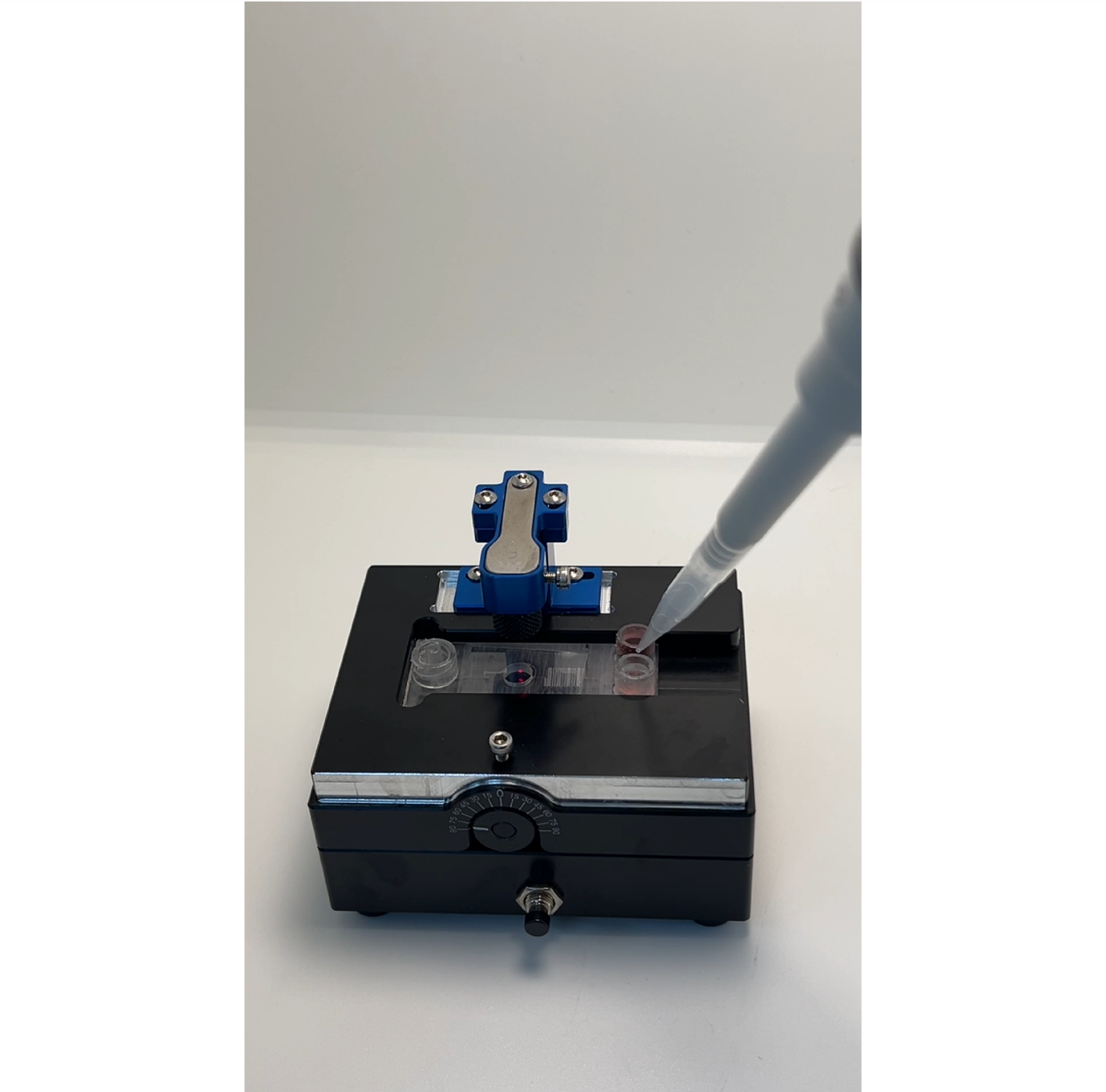


References

1. Thermo Fisher Scientific. Human SARS-CoV-2 Spike (Trimer) Ig Total ELISA Kit - Invitrogen. https://www.thermofisher.com/elisa/product/Human-SARS-CoV-2-Spike-Trimer-Ig-Total-ELISA-Kit/BMS2323.

2. Human Thrombin ELISA Kit - Invitrogen. *Thermo Fisher Scientific* https://www.thermofisher.com/elisa/product/Human-Thrombin-ELISA-Kit/EEL053.

3. Corman, V. M. *et al.* Detection of 2019 novel coronavirus (2019-nCoV) by real-time RT-PCR. *Eurosurveillance* **25**, (2020).

4. Chan, J. F.-W. *et al.* Improved Molecular Diagnosis of COVID-19 by the Novel, Highly Sensitive and Specific COVID-19-RdRp/Hel Real-Time Reverse Transcription-PCR Assay Validated *In Vitro* and with Clinical Specimens. *J Clin Microbiol* **58**, e00310-20 (2020).

5. Abbott Diagnostics. BinaxNOW^TM^ COVID-19 Ag test card. https://www.globalpointofcare.abbott/us/en/product-details/binaxnow-covid-19.html.

6. Jiang, J., Cai, Q. & Deng, M. Construction of Electrochemical Aptamer Sensor Based on Pt-Coordinated Titanium-Based Porphyrin MOF for Thrombin Detection. *Front. Chem.* **9**, 812983 (2022).

7. Kotlarek, D. *et al.* Compact Grating-Coupled Biosensor for the Analysis of Thrombin. *ACS Sens.* **4**, 2109–2116 (2019).

8. Kotlarek, D. *et al.* Surface plasmon resonance-based aptasensor for direct monitoring of thrombin in a minimally processed human blood. *Sensors and Actuators B: Chemical* **320**, 128380 (2020).

9. Inoue, S. *et al.* A reliable aptamer array prepared by repeating inkjet-spotting toward on-site measurement. *Biosensors and Bioelectronics* **85**, 943–949 (2016).

10. Dillen, A., Mohrbacher, A. & Lammertyn, J. A Versatile One-Step Competitive Fiber Optic Surface Plasmon Resonance Bioassay Enabled by DNA Nanotechnology. *ACS Sens.* **6**, 3677–3684 (2021).

11. Liu, X. *et al.* Target-Induced Conjunction of Split Aptamer Fragments and Assembly with a Water-Soluble Conjugated Polymer for Improved Protein Detection. *ACS Appl. Mater. Interfaces* **6**, 3406–3412 (2014).

12. Cennamo, N. *et al.* SARS-CoV-2 spike protein detection through a plasmonic D-shaped plastic optical fiber aptasensor. *Talanta* **233**, 122532 (2021).
